# Ambulance Services Associated with Extreme Temperatures and Fine Particles in a Subtropical Island

**DOI:** 10.1038/s41598-020-59294-8

**Published:** 2020-02-18

**Authors:** Yu-Chun Wang, Yu-Kai Lin, Yi-Jhih Chen, Shih-Chan Hung, Yasmin Zafirah, Fung-Chang Sung

**Affiliations:** 1 Department of Environmental Engineering, College of Engineering, Chung Yuan Christian University, 200 Chung-Pei Road, Zhongli, 320 Taiwan; 2 Department of Health and Welfare, University of Taipei College of City Management, 101 Zhongcheng Road Sec. 2, Taipei, 111 Taiwan; 3 Department of Emergency Services, Ministry of Health and Welfare Nantou Hospital, Nantou, 540 Taiwan; 4 Department of Health Services Administration, China Medical University, 91 Hsueh-Shih Road, Taichung, 404 Taiwan; 5 Management Office for Health Data, China Medical University Hospital, Taichung, 404 Taiwan; 6 Department of Food Nutrition and Health Biotechnology, Asia University, Taichung, 413 Taiwan

**Keywords:** Public health, Risk factors

## Abstract

This study evaluated the association between the risk of events requiring ambulance services and the ambient temperature and particulate matter of 2.5 μm (PM_2.5_) and 10 μm (PM_10_) for populations living in subtropical Taiwan. We used a distributed lag nonlinear model with a quasi-Poisson function to assess the roles of ambient temperature, PM_10_ and PM_2.5_ in the use of ambulance services for respiratory distress, coma and unconsciousness, chest pain, lying down in public, headaches/dizziness/vertigo/fainting/syncope and out-of-hospital cardiac arrest (OHCA). The relative risk (RR) and 95% confidence interval (CI) of each specific event were calculated in association with the ambient conditions. In general, the events that required ambulance services had a V-shaped or J-shaped association with the temperature, where the risks were higher at extreme temperatures. The RR of each event was significant when the patients were exposed to temperatures in the 5^th^ percentile (<15 °C); patients with OHCA had the highest adjusted RR of 1.61 (95% CI = 1.47–1.77). The risks were also significant for coma/unconsciousness, headaches/dizziness/vertigo/fainting/syncope, and OHCA but not for respiratory distress, chest pain and lying down in public, after exposure to the 99^th^ percentile temperatures of >30 °C. The risks for use of ambulance services increased with PM exposure and were significant for events of respiratory distress, chest pain and OHCA after exposure to the 99^th^ percentile PM_2.5_ after controlling for temperatures. Events requiring ambulance services were more likely to occur when the ambient temperature was low than when it was high for the population on the subtropical island of Taiwan. The association of the risk of events requiring ambulance services with PM were not as strong as the association with low temperatures.

## Introduction

The increased frequency and intensity of extreme climate events are important public health concerns^1^. Studies have reported that ambient temperature and air pollution are important factors with significant impacts associated with various morbidities and mortalities^2–4^. The associations between mortality and the temperature have been characterized by U-shaped, V-shaped and J-shaped curves^5,6^, with mortality increasing at extremely cold and/or extremely hot temperatures^7,8^. Studies have also found that the risk of emergency room visits for out-of-hospital cardiac arrest (OHCA) was greater in cold seasons than in hot seasons^9–11^. In Taiwan, the cumulative 6-day relative risk of emergency room visits for OHCA reaches 1.73 when the mean temperature is 14 °C in comparison to when it is >27 °C after controlling for air pollution^12^.

Climate conditions have important impacts on the transport and dispersion of air pollutants. The role of pollutants in health impacts may thus vary with climate conditions^13^. However, studies may emphasize the impacts of air pollution rather than temperature on health, especially for respiratory diseases. One study that used the Danish Diet, Cancer, and Health cohort to follow 53,695 adults aged 50–65 years for a median of 10.2 years^14^ found that NO_2_ exposure increased the risk of hospitalization for asthma and chronic obstructive pulmonary disease but not for stroke. No climate factors were considered in the Danish study. Cheng *et al*.^15^ also found that the air pollution in Kaohsiung, Taiwan, increased the risk of hospital admission for pneumonia in people with upper respiratory infections. The impact was found to be greater on cold days than on warm days, indicating that the climate played a role. Pneumonia is a significant consequence of influenza and cold symptoms, which appear mainly in the cold season.

Studies found that the ambulance dispatch services in Italy and Japan increased due to emergency events of OHCA, respiratory disorders and chest pain, etc., during a period with extreme temperatures^16,17^. A recent study found a 2-fold increase in the use of emergency ambulance services in Taiwan within a 10-year period^18^. In contrast to visits for nonurgent conditions, which mostly occur at hospital emergency departments^19^, the number of ambulance emergency calls may be attributed to complicated conditions including delayed arrival times, restricted service times, and hard-to-reach locations. Moreover, a call is considered to be covered if it is responded to within a predefined standard time of 10 minutes in an urban area^20^. Most citizens use public ambulance systems as their initial entry points for receiving urgent care^21^. Ambulance call-out data provide new and valuable real-time information that is useful for assessing the impacts of environmental conditions, such as temperature and air pollution, on human health^22^.

Taiwan is a 150 km wide and 350 km long subtropical island within a relatively narrow range of longitude and latitude (22–25°N, 120–122°E) and an annual average temperature of 24 °C, which varies from north to south^23^. The mean daily temperatures in urban areas range from 8 °C in the winter to 33 °C in the summer. The present study evaluated the relationship between the use of ambulance services and the ambient temperature conditions and fine particulate matter of 2.5 μm and 10 μm (PM_2.5_ and PM_10_).

## Materials and Methods

### Data Sources

We obtained the ambulance services data from the Ministry of Health and Welfare, meteorological data from the Central Weather Bureau (CWB) and hourly air pollution monitoring records from the Environmental Protection Administration (EPA) of Taiwan, all from 2006 to 2014. The ambulance services database contained information on the gender and age of the patient, the reason for the ambulance care call, and the location and time the service was dispatched for the event. All personal identification had been scrambled into surrogate numbers for privacy protection before the data file was released to users. From the medical records in the ambulance services data, the patients diagnosed with respiratory distress, coma and unconsciousness, chest pain, headaches/dizziness/vertigo/fainting/syncope, lying down in public, and out-of-hospital cardiac arrest in 15 cities and counties in Taiwan were identified, with the exception of the Taipei metropolitan area, which is the most urbanized area in Taiwan. The daily cause-specific number of these events requiring ambulance services in the area were analyzed for associations with the ambient environmental conditions.

**Figure 1:**
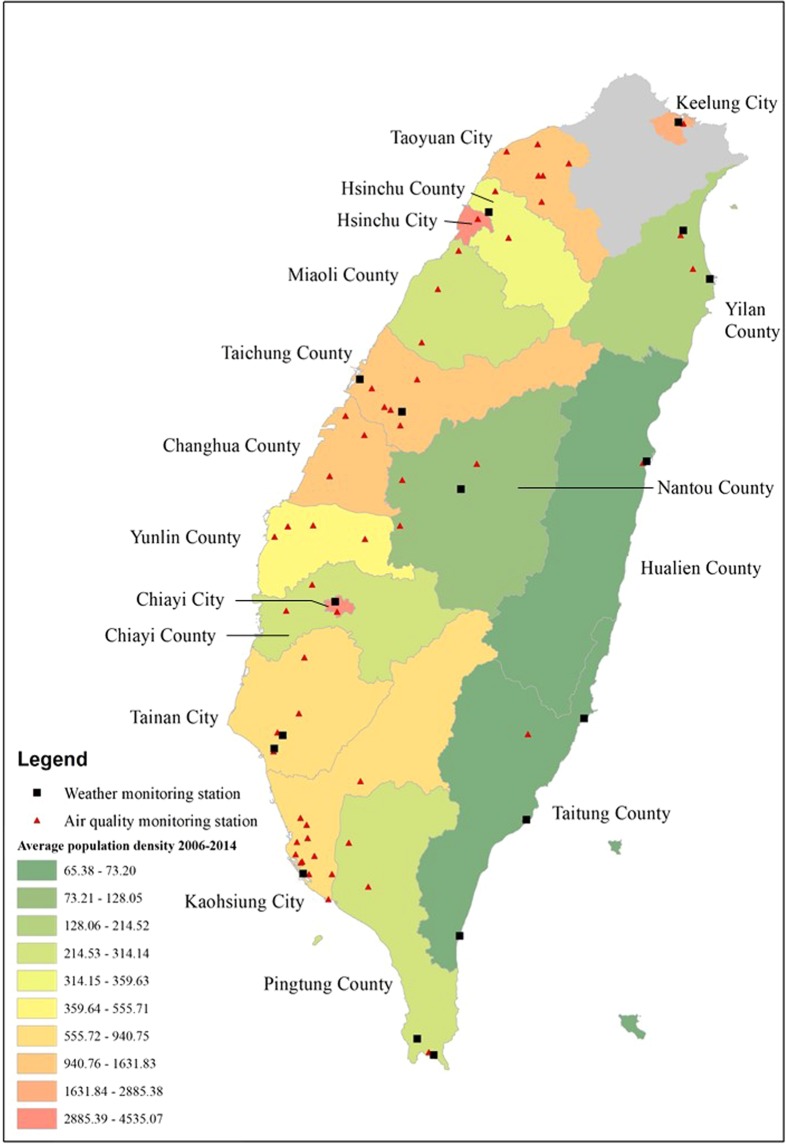
Locations of weather observatories and ambient air quality monitor stations.

The CWB meteorological records consisted of hourly weather data, which included the average temperature (°C), maximum temperature (°C), minimum temperature (°C), relative humidity (%), wind speed (m/s), and barometric pressure, that were monitored at 25 real-time surface meteorological observatories around Taiwan. For areas without observatory stations, such as Taoyuan, Miaoli, Changhua, Yunlin, and Nantou, the weather data were obtained from the nearest surface meteorological observatory (Fig. 1).

The Taiwan air quality monitoring network has been in operation since 1993 and provides hourly records of ambient air pollutants, such as PM_10_, sulfur dioxide, nitrogen dioxide (NO_2_), ozone, and carbon monoxide measured at the 76 stationary monitoring stations distributed throughout the island (Fig. 1). PM_2.5_ has been monitored since 2006. Detailed information on the monitoring instruments, stations, and quality assurance criteria are available through the Taiwan EPA website (https://taqm.epa.gov.tw/taqm/en/default.aspx).

### Statistical Models

#### Nonlinear association between the daily ambient environmental and health risks

The daily means and ranges of the ambient environmental characteristics and the numbers of patients receiving ambulance services by the cause of the event from 2006 to 2014 were calculated for all regions of Taiwan. This study adopted a distributed lag nonlinear model (DLNM) with a quasi-Poisson function, as proposed by Gasparrini *et al*.^24^, to assess the nonlinear exposure-response relationship and a delayed association between the daily ambient environment and the cause-specific number of events requiring ambulance services, which was specified as follows:

1 $$\begin{array}{c}\mathrm{Log}[{\rm{Y}}] \sim {\rm{BS}}({\rm{T}},\,{\rm{lag}}=3\,{\rm{day}})+{\rm{BS}}({{\rm{PM}}}_{2.5}{\rm{or}}\,{{\rm{PM}}}_{10},\,{\rm{lag}}=5\,{\rm{day}})\\ \,+{\rm{NS}}({{\rm{NO}}}_{2},4)+{\rm{NS}}({\rm{date}},4\,{\rm{per}}\,{\rm{year}})+{\rm{NS}}({\rm{ws}},4)+{\rm{NS}}({\rm{rh}},4)\\ \,+{\rm{holiday}}\,{\rm{effect}}+{\rm{dow}}+{\rm{PI}}\end{array}$$

where *Y* is the cause-specific daily number of events requiring ambulance services in a specific area, and *T* is the area-specific daily mean temperature. The reference temperature was set at 25 °C for all categories. This temperature was selected because we observed that it was the temperature with the lowest frequency of ambulance calls. We used the basis spline (*BS*) function with 4 degrees of freedom (d*f*) for the daily average temperature to estimate the association between the temperature and the health risk, and the effects were estimated and accumulated for 4 days. Most studies accumulate the lag effects of the ambient temperature on morbidity from a lag of 0 to a lag of 3^25^. The area-specific daily PM_10_ and PM_2.5_ concentrations were included in the model and set with 4 *df*. The daily NO_2_ concentration, wind speed (ws) and relative humidity (rh) were included in the model and set in the natural spline (*NS*) function with 4 d*f*. *dow* indicates the day of the week. The daily deaths from pneumonia and influenza (PI) were also included in the model. The model selection was based on the lower Akaike information criterion value.

The relative risk (RR) and the related 95% confidence interval (CI) calculations for each cause of an emergency ambulance service event that was associated with extreme temperatures in the 5^th^ and 99^th^ percentiles relative to 25 °C were controlled for the PM and NO_2_ concentrations, wind speed, relative humidity, holiday effect, day of a week, mortality from pneumonia and influenza, and long-term time trend. We further calculated the relative risk of each cause by the increase of PM_10_ relative to 40 μg/m^3^ PM_10_ (Q1) and by the increase of PM_2.5_ relative to 20 μg/m^3^ PM_2.5_ (Q1), without and with controlling for temperature.

#### Meta-analysis

We calculated the area-specific relative risk and the 95% CI of the cause-specific ambulance events for each county and city in association with the temperature (Supplementary Figs. S1–S6) and the PM_10_ (Supplementary Figs. S7–S12) and PM_2.5_ concentrations (Supplementary Figs. S13–S18). We further integrated the relative risk of each ambulance services event for all areas into a relative risk of the ambulance services events for the whole Taiwan area through multivariate meta-analysis. The reference temperature was also set at 25 °C, which was the temperature related to the lowest ambulance call risk. The meta-analysis was fitted using a random-effects model with maximum likelihood^26^. The heterogeneity was evaluated using a multivariate extension of the *I*^2^ value, where the values (ranging from 0–100%) increased with increasing heterogeneity. All analyses in this study were carried out using the *mgcv*, *dlnm*, and *mvmeta* packages in R version 3.4.0.

## Results

### Climate characteristics and trends in the use of ambulance services from 2006 to 2014

During the study period, the island-wide daily mean temperature was 23.4 °C (range: 10.6–31.0 °C), with a mean relative humidity of 76.7% (range: 53.9–93.0%), wind speed of 2.56 m/s (range: 1.14–9.84 m/s), NO_2_ concentration of 14.6 *μg*/m^3^ (range: 3.32–35.8 *μg*/m^3^), PM_10_ concentration of 54.0 *μg*/m^3^ (range: 17.4–372 *μg*/m^3^) and PM_2.5_ concentration of 30.2 *μg*/m^3^ (range: 6.65–106 *μg*/m^3^) (Table 1). The mean daily numbers of cases requiring ambulance services were 79.6 for headaches/dizziness/vertigo/fainting/syncope, 53.4 for coma and unconsciousness, 63.0 for respiratory distress, 27.1 for lying down in public, 31.2 for chest pain, and 30.5 for OHCA.

**Table 1:** Characteristics of daily ambient environment conditions and daily cause-specific cases cared by ambulance services in Taiwan from 2006 to 2014.

Parameters	Mean	S.D.	Minimum	Q1	Q2	Q3	Maximum
**Ambulance Services**							
Respiratory distress	63.0	18.7	22.0	48.0	62.0	76.0	149
Coma and unconsciousness	53.4	16.8	16.0	40.0	53.0	65.0	125
Chest pain	31.2	10.8	6.00	23.0	31.0	39.0	65.0
Headache/dizziness/vertigo/fainting/syncope	79.6	20.4	33.0	64.0	78.0	94.0	168
Lying in public	27.1	7.43	7.00	22.0	27.0	32.0	55.0
Out-of-hospital cardiac arrest	30.5	12.1	0	22.0	30.0	38.0	76.0
**Vital statics**							
Pneumonia and influenza	17.9	5.94	2	14.0	17.0	22.0	44.0
**Environmental factors**							
Temperature (°C)	23.4	4.60	10.6	19.6	24.2	27.5	31.0
Relative humidity (%)	76.7	5.71	53.9	73.5	77.1	80.5	93.0
Wind speed (m/s)	2.56	0.88	1.14	1.93	2.31	2.97	9.84
PM_10_ (μg/m^3^)	54.0	22.7	17.4	37.1	49.9	66.7	372
PM_2.5_ (μg/m^3^)	30.2	13.3	6.65	19.9	27.9	38.2	106
NO_2_ (ppb)	14.6	4.23	3.32	11.0	14.2	17.4	35.8

**Figure 2:**
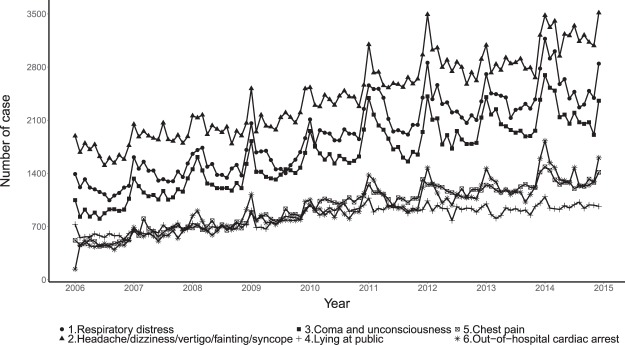
Monthly trends of cause-specific ambulance services from 2006 to 2014 in Taiwan.

Figure 2 shows the monthly cause-specific events that received ambulance services for the whole population of Taiwan; most types of incidents tended to increase from 2006 to 2014 except for OHCA. Annual peaks usually occurred in December and January during the cold season. The event type with the highest incidence was headaches/dizziness/vertigo/fainting/syncope, followed by respiratory distress, coma and unconsciousness, and chest pain. Supplementary Table 1 lists descriptive statistic for age and sex stratified case number from 2006 to 2014 in Taiwan except Taipei and New Taipei city.

### Relative risk of events requiring ambulance services in relation to the ambient temperature and levels of PM_10_ and PM_2.5_

**Figure 3:**
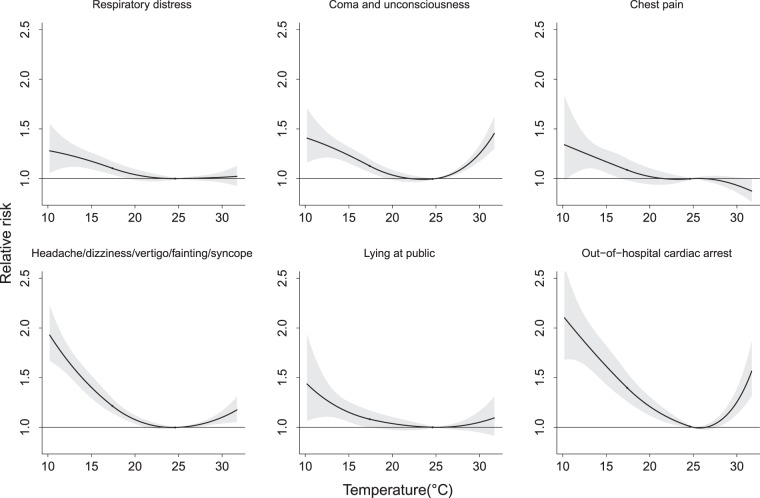
Relative risk of cause-specific disorders for ambulance ervices associated with daily average temperature relative to 25 °C by meta-analysis.

Figure 3 shows that the relative risks of the cause-specific events requiring ambulance services were mainly v-shaped in relation to the daily mean temperature for all areas across Taiwan. Compared with the temperature of 25 °C, the risk was greater at low temperatures than at high temperatures, with a relative risk near 2.0 for the incidence of headaches/blackouts/fainting/syncope and OHCA when the temperature was <10 °C. A completely inverse relationship appeared between the incidence of chest pain and the ambient temperatures. Fig. S6 illustrates the area-specific relationships between OHCA and temperature. An elevated RR for OHCA was observed in most areas when the temperature was low, mainly in Taoyuan, Hsincu, Miaoli, Taichung, Chunghua, Nantou, Yunlin, Tainan, Kaohsiung, Pingtung, and Yilan.

The cause-specific RRs for the use of ambulance services associated with extreme temperatures in the 5^th^ and 99^th^ percentile are presented in Table 2. All 6 types of ambulance services were significantly elevated in the low-temperature environment (<15 °C), with the highest risk found for OHCA (RR = 1.61; 95% CI: 1.47–1.77). However, the risks were not significant at the extremely high temperatures of >30 °C for respiratory distress (RR = 1.01; 95% CI: 0.93–1.09) or chest pain (RR = 0.91; 95% CI: 0.82–1.01).

**Table 2:** Cause-specific relative risk (95% confidence interval) of ambulance services associated with the 5^th^ and 99^th^ percentile extreme temperatures.

	Tavg. at 5^th^ percentile (15 °C)		Tavg. at 99^th^ percentile (31 °C)	
	RR	95% CI	RR	95% CI
Respiratory distress	1.15	(1.08–1.22)	1.01	(0.93–1.09)
Coma and unconsciousness	1.23	(1.16–1.31)	1.34	(1.23–1.46)
Chest pain	1.14	(1.05–1.25)	0.91	(0.82–1.01)
Headache/dizziness/vertigo/fainting/syncope	1.41	(1.30–1.53)	1.13	(1.04–1.22)
Lying in public	1.14	(1.04–1.23)	1.09	(0.95–1.26)
Out-of-hospital cardiac arrest	1.61	(1.47–1.77)	1.40	(1.25–1.57)

RR (95% CI), relative risk (95% confidence interval) estimated after controlling for PM2.5 and NO2 concentrations, wind speed, relative humidity, holiday effect, day of a week, mortality from pneumonia and influenza, and long-term time trend.

**Figure 4:**
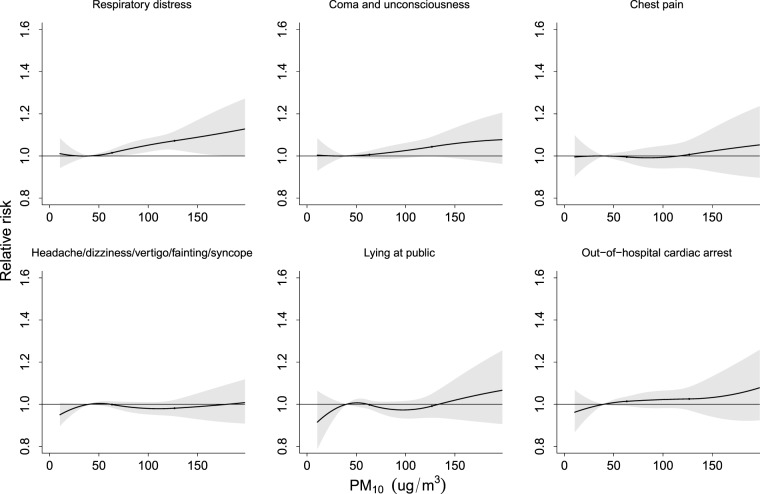
Relative risk of cause-specific disorders for ambulance services associated with daily PM_10_ concentrations relative to 40 μg/m^3^ level by meta-analysis after temperature adjusted.

**Figure 5:**
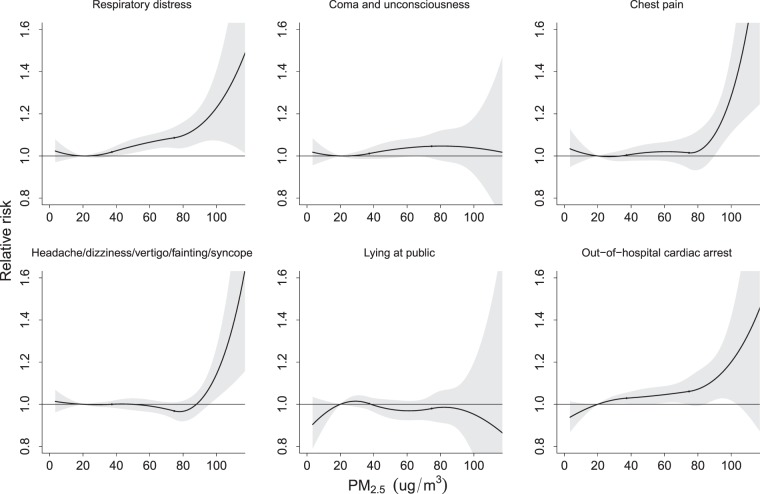
Relative risk of cause-specific disorders for ambulance services associated with daily PM_2.5_ concentrations relative to at 20 μg/m^3^ level by meta-analysis after temperature adjusted.

Figure 4 illustrates that the cause-specific RRs for the use of ambulance services increased with the daily mean PM_10_ concentration but were not significant. However, significant risks appeared for chest pain, headaches/blackouts/fainting/syncope and OHCA when the PM_2.5_ concentration reached approximately 90 μg/m^3^ (Fig. 5).

The cause-specific relative risks of events requiring ambulance services associated with the daily 99^th^ percentile of PM_10_ (155 μg/m^3^) and PM_2.5_ (91 μg/m^3^) relative to the Q1 levels (40 μg/m^3^ and 20 μg/m^3^, respectively) are shown in Table 3. After controlling for temperature, significant risks were observed for respiratory distress (RR = 1.15; 95% CI: 1.07–1.24), chest pain (RR = 1.11; 95% CI: 1.01–1.23) and OHCA (RR = 1.12; 95% CI: 1.02–1.23) when people were exposed to levels of PM_2.5_ in the 99^th^ percentile.

**Table 3:** Cause-specific relative risk (95% confidence interval) of ambulance services associated with daily 99th percentile PM_10_ (155 μg/m^3^) and PM_2.5_(91 μg/m^3^) relative to Q1 levels (40 μg/m^3^ and 20 μg/m^3^, respectively).

	Model without adjusting temperature		Model adjusted temperature	
	RR	95% CI	RR	95% CI
**Respiratory distress**				
PM_10_, 99^th^ percentile	1.05	(0.97–1.13)	1.09	(1.01–1.18)
PM_2.5_, 99^th^ percentile	1.10	(1.02–1.19)	1.15	(1.07–1.24)
**Coma and unconsciousness**				
PM_10_, 99^th^ percentile	1.01	(0.94–1.09)	1.06	(0.99–1.14)
PM_2.5_, 99^th^ percentile	0.99	(0.92–1.07)	1.04	(0.96–1.14)
**Chest pain**				
PM_10_, 99^th^ percentile	0.98	(0.88–1.10)	1.03	(0.92–1.14)
PM_2.5_, 99^th^ percentile	1.07	(0.98–1.18)	1.11	(1.01–1.23)
**Headache/dizziness/vertigo/fainting/syncope**				
PM_10_, 99^th^ percentile	0.91	(0.86–0.97)	0.99	(0.93–1.06)
PM_2.5_, 99^th^ percentile	0.93	(0.88–0.99)	1.02	(0.97–1.09)
**Lying in public**				
PM_10_, 99^th^ percentile	0.99	(0.90–1.09)	1.02	(0.93–1.13)
PM_2.5_, 99^th^ percentile	0.94	(0.84–1.04)	0.98	(0.88–1.09)
**Out-of-hospital cardiac arrest**				
PM_10_, 99^th^ percentile	0.96	(0.87–1.06)	1.03	(0.94–1.14)
PM_2.5_, 99^th^ percentile	1.01	(0.92–1.11)	1.12	(1.02–1.23)

RR (95% CI), relative risk (95% confidence interval) estimated after controlling for temperature.

## Discussion

Ours study assessed the associations between daily usage of ambulance services and the ambient environmental conditions in Taiwan. This study defined extreme heat and cold as daily mean temperatures in the 99^th^ and 5^th^ percentiles of the temperature distribution. Low temperatures (≤15 °C) significantly elevated the risks of all ambulance events, with the highest relative risk for OHCA (RR = 1.61; 95% CI: 1.47–1.77). Ambulance care for comas and unconsciousness, OHCA and headaches/dizziness/vertigo/fainting/syncope were also associated with high temperatures (≥31 °C). High levels of PM_2.5_ (approximately 90 μg/m^3^) were found to be associated with minor increases in the risks of ambulance care for respiratory distress, chest pain, and OHCA (relative risk ranges from 1.11 to 1.15).

This study focused on ambulance calls based on the data from several reports. Respiratory diseases impose a large burden worldwide, and some of these diseases are categorized as the most common causes of severe illness and death worldwide^27^. Complaints of respiratory distress account for 13% of the total emergency medical services calls in the United States^28^. Unconsciousness also represented a relatively large proportion of the group requiring emergency medical services^29^. Chest pain was selected because it is involved in a higher frequency of emergency medical services, reaching as high as 14% of calls in the United States^30^. Headache symptoms are the most common disorder and have been reported by approximately 18% of women and 6% of men in the United States^31^. El Sayed *et al*. recently reported that 60% of all emergency medical services were for OHCA in the United States^32^. Our study showed that during the study period, headaches/dizziness/vertigo/fainting/syncope was the most prevalent event (27.9%) requiring ambulance services, and lying down in the public was the least frequent event (9.52%), whereas 10.7% of the services were for OHCA.

Limited reports have disclosed associations between the number of ambulance calls and the ambient environment^16,17,33–35^. The temperature-health risk associations may vary across locations and health outcomes^36^. A study in Italy evaluated the cause and age stratification of ambulance dispatches in association with biometeorological discomfort and the apparent temperature in the region of Emilia-Romagna, which is located in northern Italy. Greater risks appeared when the daily mean apparent temperature exceeded 30 °C, and the risk increased with age^16^. In Brisbane, the demand for heat-related ambulance calls increased immediately and lasted for 24 hours when the hourly temperature was higher than 27 °C, and the relative risk reached 1.8 when the temperature was 36 °C^34^. A Huainan study in China found that extreme heat and heatwaves were significantly associated with increased emergency ambulance dispatches but that the risk may decrease with higher density and longer duration of the heatwaves^33^. In the present study, we also observed increased ambulance events when the daily mean temperature exceeded 27 °C. However, all events that required ambulance care were even more frequent when the temperature was lower than 18 °C. Studies have consistently reported that the subtropical climate in Taiwan is associated with greater health risks when temperatures are lower than when temperatures are higher^37,38^. The population in Taiwan may acclimatize to environments with higher temperatures and be sensitive to a climate with lower temperatures. Some studies have reported that the effects of cold temperatures are most significant in warm regions. People in warm regions are less adapted physically, socially, and behaviorally to low temperatures^39^. A study in Hong Kong found that the elderly were using ambulance services frequently due to their potential to suffer from cold temperatures below 12 °C, which is considered very cold in Hong Kong^40^.

This study agreed with the previous research that found PM_2.5_ to be significantly associated with respiratory tract diseases; the risk of respiratory tract diseases was greater in response to exposure to traffic-related air pollutants^41^. Chest pain was found to be significantly associated with PM_2.5_; in addition, a higher PM_2.5_ concentration may enhance the number of incidents of chest pain during warm days^42^. Kenneth *et al*. found that PM_2.5_ could trigger severe headaches by activating the sympathetic nervous system^31^.

This study showed that among the 6 categories of ambulance services, OHCA had the strongest RR in association with extreme temperatures and higher ambient levels of PM_2.5_. Studies in Japan have shown that the risk of OHCA was significantly elevated at low temperatures^9,10,35^. However, a study in Guangzhou, China, suggested that the OHCA risk increased on both cold and hot days and showed a J-shaped relationship with the temperature factors^35^, similar to the pattern in Taiwan, where the risk was higher on cold days than on hot days. Low temperatures may increase blood viscosity, plasma viscosity, arterial pressure, and plasma cholesterol concentrations, thereby intensifying the stress on the cardiovascular system^43^.

The risk of OHCA increased with increasing ambient PM levels^11,44–47^. Ozone has also been associated with OHCA, while other pollutants are less likely to be associated with OHCA^48^. A Korean study reported that the OHCA risk increased by 1.30% after 1 to 2 days of exposure to PM_2.5_, with an elevation of 10 μg/m^3^ ^44^. The impact of PM_2.5_ on OHCA has been shown from an immediate effect (a few hours or same-day exposure prior to OHCA) to a lag of 2 days^49^. The pathogenicity of PM is determined by its size. PM with aerodynamic diameters smaller than 10 µm has a greater impact on human health than PM with a larger diameter. The major components of PM_2.5_ are inorganic sulfate, organic carbon, trace elements and ammonium^50^, while PM_10_ consists of crustal dust, secondary sulfate and nitrate, metal emission source and vehicle exhaust^51^. PM_2.5_ particles are characterized by a small diameter (<2.5 µm) and are able to carry various toxic substances; they are not filtered by the nose and reach to the end of the respiratory tract with the airflow, accumulating by diffusion and damaging other parts of the body through air exchange with the lungs^52^.For the present study, the regional associations between the cause-specific ambulance services and the ambient PM_10_/PM_2.5_ concentrations are shown in Figs. S7–S18. To date, no conclusive evidence has suggested that the OHCA risk is linked to exposure to airborne pollutants. In addition to OHCA, the present study also observed some significant risks associated with higher PM concentrations, which mainly occurred in central and southern Taiwan.

Taiwan is a relatively small island, but our study demonstrated that the linkage between OHCA and temperature varied among geographic areas. Elevated OHCA risk was found with low temperatures in most study areas, except in Keelung, Chiayi, Hualien and Taitung. On the other hand, the elevated OHCA risk was also found in populations exposed to extremely high temperatures in Keelung, Miaoli, Chunghua, Chiayi, Tainan, Kaohsiung, Hualien and Taitung (Supplementary Fig. S6). These findings agreed with our previous reports, which indicated that the temperature-health association in Taiwan exhibited spatial heterogeneity^4,37,38^. The acclimatization and adaptive capacity of people to extreme temperatures likely varies among areas.

In addition to OHCA, other types of ambulance events, including respiratory distress, coma and unconsciousness, chest pain, and headaches/dizziness/vertigo/fainting/syncope, were also associated with the ambient temperatures in Taiwan, especially for the major cities, namely, Taoyuan, Tainan and Kaohsiung. Overall, there was a more significant relationship associated with lower temperatures than with higher temperatures. Our data agreed with those from a previous study, which reported that people living in warm climates were generally more vulnerable to cold^23^. The connection of these general symptoms with the development of specific diseases and the mechanisms associated with the ambient temperatures require future research.

The association between temperature and PM_10_ and PM_2.5_ and mortality and morbidity has been reported in several studies^53,54^. A study in Brisbane and Italy found that the health effect of PM_10_ increased on warmer days^53,54^, while a study in Hong Kong discovered a greater effect of PM_2.5_ on respiratory mortality in low temperature ranges than in high temperature ranges^55^. The present study showed that the relative risks of respiratory distress, chest pain, and OHCA among ambulance services associated with daily PM_10_ and PM_2.5_ in the 99^th^ percentile increased after controlling for temperature (Table 3). In addition, the association of daily PM_10_ and PM_2.5_ in the 75^th^ percentile with the use of ambulance services has been analyzed. The risk was only significant for respiratory distress and OHCA. Although the mechanism of the interaction between air pollution and temperature remains unclear, some possible explanations have been proposed. The effect of PM_2.5_ with high temperatures may be linked to the direct or indirect responses of organisms to heat stress. Low temperatures may cause physiologic stress, thus reducing the physiologic response to air pollution; as a result, people are more susceptible to air pollution^55,56^. In cold seasons, particularly with extremely low temperatures in the 5^th^ percentile, temperature inversion occurs and consequently exacerbates the accumulation of PM_10_ and PM_2.5_. We should take note of the confounding characteristics of PM exposure.

The associations between the ambient temperature and health indicators, such as mortality, emergency room visits and outpatient visits, are varied due to population variations in health status, socioeconomics, and access to medical services^36^. The amount of time from exposure to the ambient environment to the development of health events also varies with health indicators. The information on this topic remains unclear due to limited population-based studies worldwide. The progression of disease, from early symptoms, outpatient visits, emergency room visits and ambulance services to death, represents the various patterns for disease severity associated with risk factors. Identifying the risks in an earlier stage of disease may provide critical information for population health interventions and policy planning for future medical services.

The present study has several strengths. The wide data coverage from the Taiwan ambulance services program and all ambient air quality data ensures the representativeness of exposure for the entire population. The confounding factors, such as the holiday effect, day of the week, long-term trends, and risks associated with infectious pneumonia and influenza, were considered in the data analysis models.

Despite the contributions of this study, there were some limitations. First, our work was an ecological study. The risk was not estimated with individual-based data. In addition, information on some factors that may modify the risk associated with ambulance events, such as smoking, drinking, exercise and socioeconomic status, was not available for evaluation^57–59^. The hourly measurements of the ambient environment associated with cause-specific ambulance services require further investigation to control for these factors. The patient diagnoses in the medical records of the ambulance services were based on observers conducting the services. No ICD codes were provided.

The rapid increase in the demand for ambulance services in Taiwan with extremely high and low temperatures and air pollution events is a critical issue that should be the focus of the government. Our current study reports that extreme temperatures play a more important role than air pollution in increasing the number of ambulance events. Therefore, our research suggests that the public health sector should take several actions, such as examining ambulance calls, especially during extremely cold temperatures and summer activities; providing medical preparation in critical conditions; and providing educational campaigns for the community to increase their awareness.

## Conclusions

This population-based study evaluated the association of events requiring ambulance services, including respiratory distress, coma and unconsciousness, chest pain, headaches/dizziness/vertigo/fainting/syncope, lying down in public, and OHCA, with ambient environmental conditions in Taiwan. The population of the island had a higher risk of OHCA in response to exposure to cold temperatures than in response to hot temperatures, although only 10.7% of ambulance services were for OHCA. In addition, ambulance care for coma and unconsciousness and headaches/dizziness/vertigo/fainting/syncope were also elevated in extreme heat exposure. The high level of PM may slightly increase the frequency of emergency medical care for respiratory distress, chest pain, and OHCA after controlling for temperature. Among the studied ambulance events, the incidence of OHCA was the most vulnerable to extreme temperatures and higher levels of PM_2.5_. This study provides critical information to health authorities for the development of future ambulance service plans.

## Supplementary information

Supplementary data.
