# Supplementary material for: Ambulance Services Associated with Extreme Temperatures and Fine Particles in a Subtropical Island
Source: Sci Rep. 2020 Feb 18;10:2855. doi: 10.1038/s41598-020-59294-8 (PMC7029034; doi:10.1038/s41598-020-59294-8)
Supplement: Supplementary file 1 — Supplementary data. [file 41598_2020_59294_MOESM1_ESM.pdf]

Title Page

Ttile : Ambulance Services Associated with Extreme Temperatures and Fine Particles in a Subtropical Island

Authors : Yu-Chun Wang, PhD, Yu-Kai Lin, PhD, Yi-Jhih Chen, Shih-Chan Hung, Yasmin Zafirah, Fung-Chang Sung

Note : The material contained herein is supplementary to the Scientific Reports.

Supplementary Table legends

Supplementary Table 1. Descriptive statistic for age and sex stratified case number from 2006 to 2014 in Taiwan except Taipei and New Taipei city

Type	Respiratory distress	Coma and unconsciousness	Chest pain	Headache/ dizziness /vertigo/ fainting/ syncope	Lying in public	Out-of-hospital cardiac arrest
**Male**	137534	104704	74482	161021	85061	74288
**Female**	101403	97686	44384	140937	15799	43200
**40-44 yr**	9741	7786	8575	16816	11934	4353
**45-49 yr**	11373	9003	9797	20174	12697	5428
**50-54 yr**	14181	12185	11813	24775	16704	7506
**55-59 yr**	13651	11693	10601	23678	10083	7511
**60-64 yr**	15401	14096	10527	25222	9847	9218
**65-69 yr**	14631	13922	8314	21425	4705	8521
**70-74 yr**	20412	21505	8995	28110	4595	12302
**75-79 yr**	24667	25062	9045	28625	3510	14310
**80-84 yr**	32601	30587	10685	29597	3644	17578
**<40 yr**	42711	22230	21305	59281	20541	8541
**>=85 yr**	39568	34321	9209	24255	2600	22220

Supplementary Figure legends

Supplementary Figure S1. Area-specific relative risk for ambulance services of respiratory distress in association with ambient daily average temperature (°C) from 2006 to 2014

Supplementary Figure S2. Area-specific relative risk for ambulance services of coma and unconsciousness in association with ambient daily average temperature (°C) from 2006 to 2014

Supplementary Figure S3. Area-specific relative risk for ambulance services of chest pain in association with ambient daily average temperature (°C) from 2006 to 2014

Supplementary Figure S4. Area-specific relative risk for ambulance services of headache/dizziness/vertigo/fainting/syncope in association with ambient daily average temperature (°C) from 2006 to 2014

Supplementary Figure S5. Area-specific relative risk for ambulance services of lying at public in association with ambient daily average temperature (°C) from 2006 to 2014

Supplementary Figure S6. Area-specific relative risk for ambulance services of out-of-hospital cardiac arrest in association with ambient daily average temperature (°C) from 2006 to 2014

Supplementary Figure S7. Area-specific relative risk for ambulance services of respiratory distress in association with ambient PM_{10} concentration ($\mu g/m^3$) from 2006 to 2014

Supplementary Figure S8. Area-specific relative risk for ambulance services of coma and unconsciousness in association with ambient PM_{10} concentration ($\mu g/m^3$) from 2006 to 2014

Supplementary Figure S9. Area-specific relative risk for ambulance services of chest pain in association with ambient PM_{10} concentration ($\mu g/m^3$) from 2006 to 2014

Supplementary Figure S10. Area-specific relative risk for ambulance services of headache/dizziness/vertigo/fainting/syncope in association with ambient PM_{10} concentration ($\mu g/m^3$) from 2006 to 2014

Supplementary Figure S11. Area-specific relative risk for ambulance services of lying at public in association with ambient PM_{10} concentration ($\mu g/m^3$) from 2006 to 2014

Supplementary Figure S12. Area-specific relative risk for ambulance services of out-of-hospital cardiac arrest in association with ambient PM_{10} concentration ($\mu g/m^3$) from 2006 to 2014

Supplementary Figure S13. Area-specific relative risk for ambulance services of respiratory distress in association with ambient $PM_{2.5}$ concentrations ($\mu g/m^3$) from 2006 to 2014

Supplementary Figure S14. Area-specific relative risk for ambulance services of coma and unconsciousness in association with ambient $PM_{2.5}$ concentration ($\mu g/m^3$) from 2006 to 2014

Supplementary Figure S15. Area-specific relative risk for ambulance services of chest pain in association with ambient $PM_{2.5}$ concentration ($\mu g/m^3$) from 2006 to 2014

Supplementary Figure S16. Area-specific relative risk for ambulance services of headache/dizziness/vertigo/fainting/syncope in association with ambient $PM_{2.5}$ concentration ($\mu g/m^3$) from 2006 to 2014

Supplementary Figure S17. Area-specific relative risk for ambulance services of lying at public in association with ambient $PM_{2.5}$ concentrations ($\mu g/m^3$) from 2006 to 2014

Supplementary Figure S18. Area-specific relative risk for ambulance services of out-of-hospital cardiac arrest in association with ambient $PM_{2.5}$ concentrations ($\mu g/m^3$) from 2006 to 2014

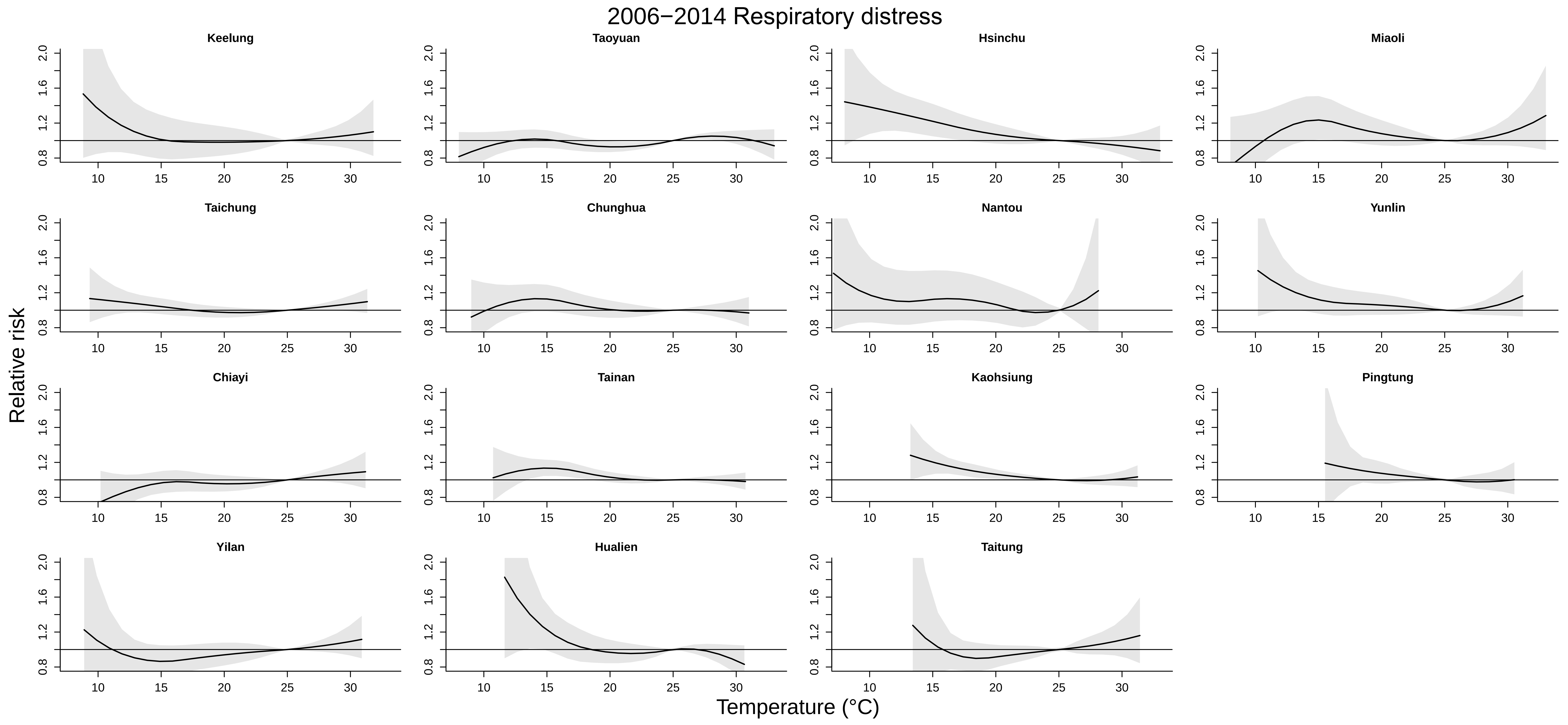

2006−2014 Respiratory distress
Keelung
Taoyuan
Hsinchu
Miaoli
Taichung
Chunghua
Nantou
Yunlin
Chiayi
Tainan
Kaohsiung
Pingtung
Yilan
Hualien
Taitung
Relative risk
Temperature (°C)
0.8
1.2
1.6
2.0
10
15
20
25
30

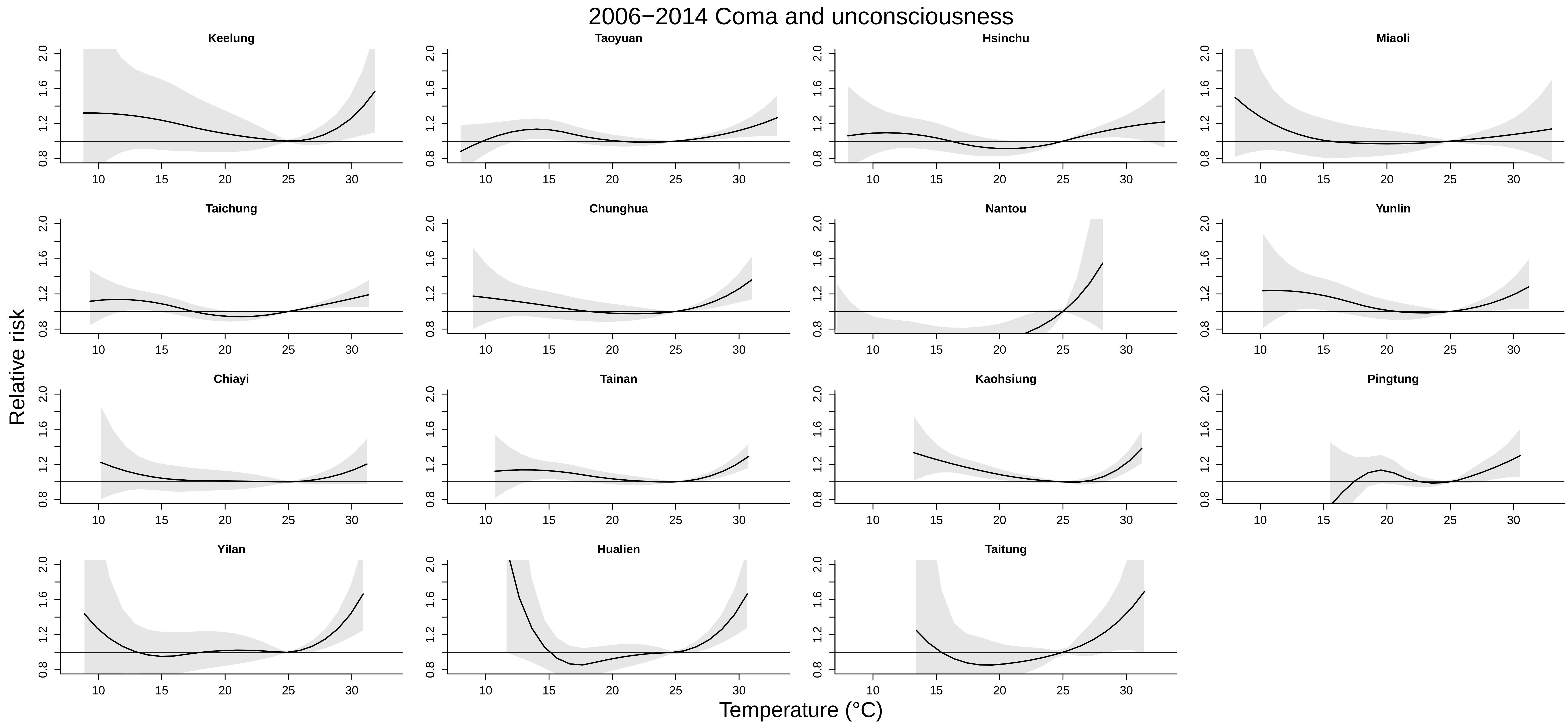
2006−2014 Coma and unconsciousness
Keelung
Taoyuan
Hsinchu
Miaoli
Taichung
Chunghua
Nantou
Yunlin
Chiayi
Tainan
Kaohsiung
Pingtung
Yilan
Hualien
Taitung
Relative risk
Temperature (°C)

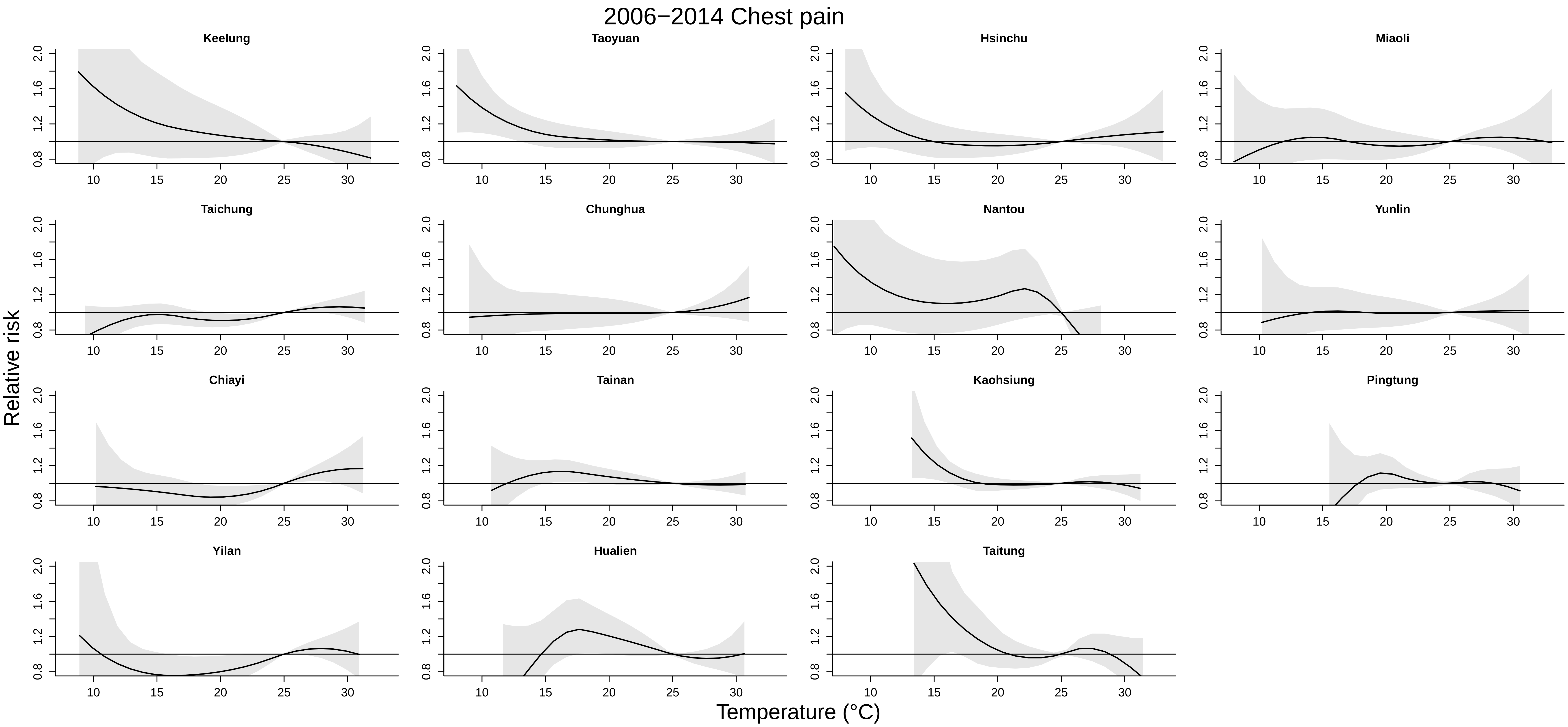

2006−2014 Chest pain
Keelung
Taoyuan
Hsinchu
Miaoli
Taichung
Chunghua
Nantou
Yunlin
Chiayi
Tainan
Kaohsiung
Pingtung
Yilan
Hualien
Taitung
Relative risk
Temperature (°C)
0.8
1.2
1.6
2.0
10
15
20
25
30

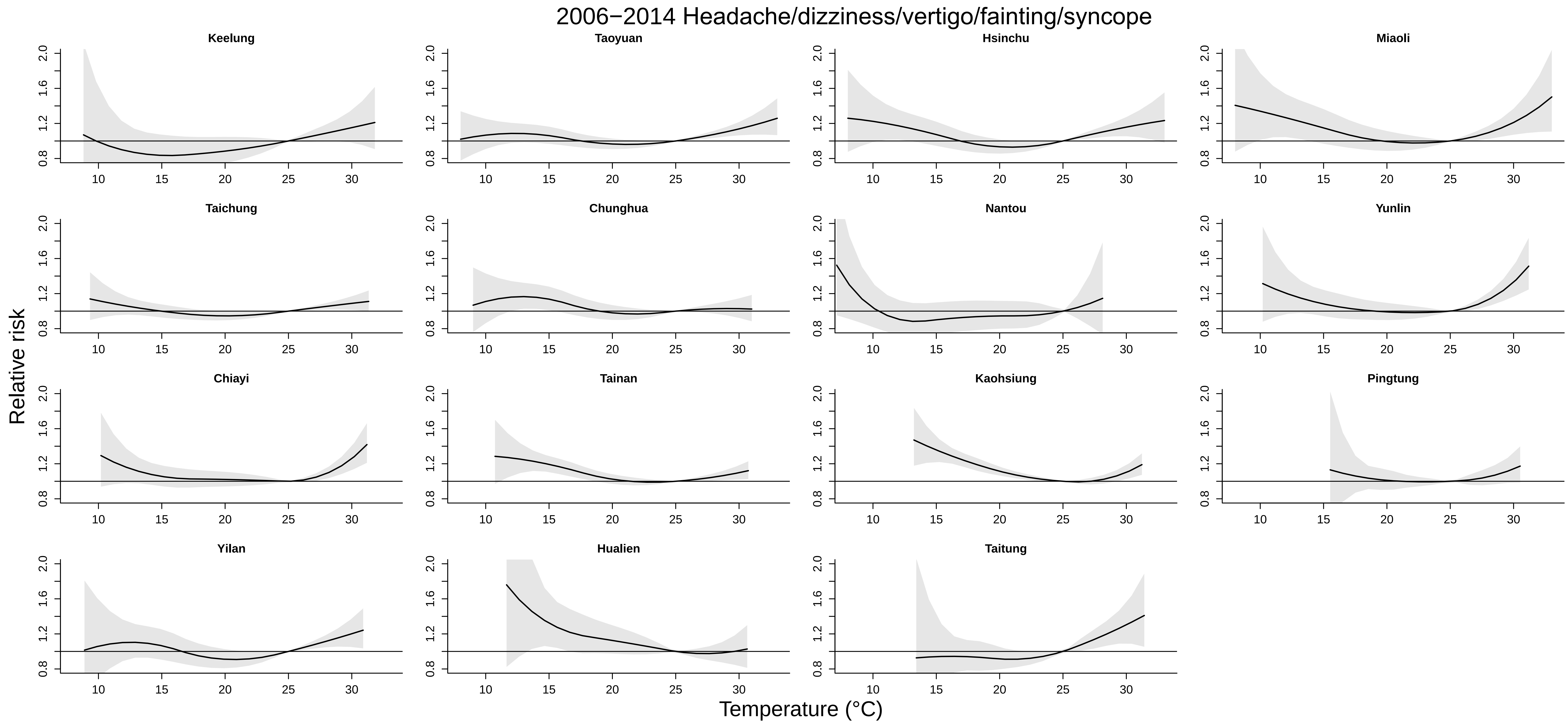
2006−2014 Headache/dizziness/vertigo/fainting/syncope
Keelung
Taoyuan
Hsinchu
Miaoli
Taichung
Chunghua
Nantou
Yunlin
Chiayi
Tainan
Kaohsiung
Pingtung
Yilan
Hualien
Taitung
Relative risk
Temperature (°C)

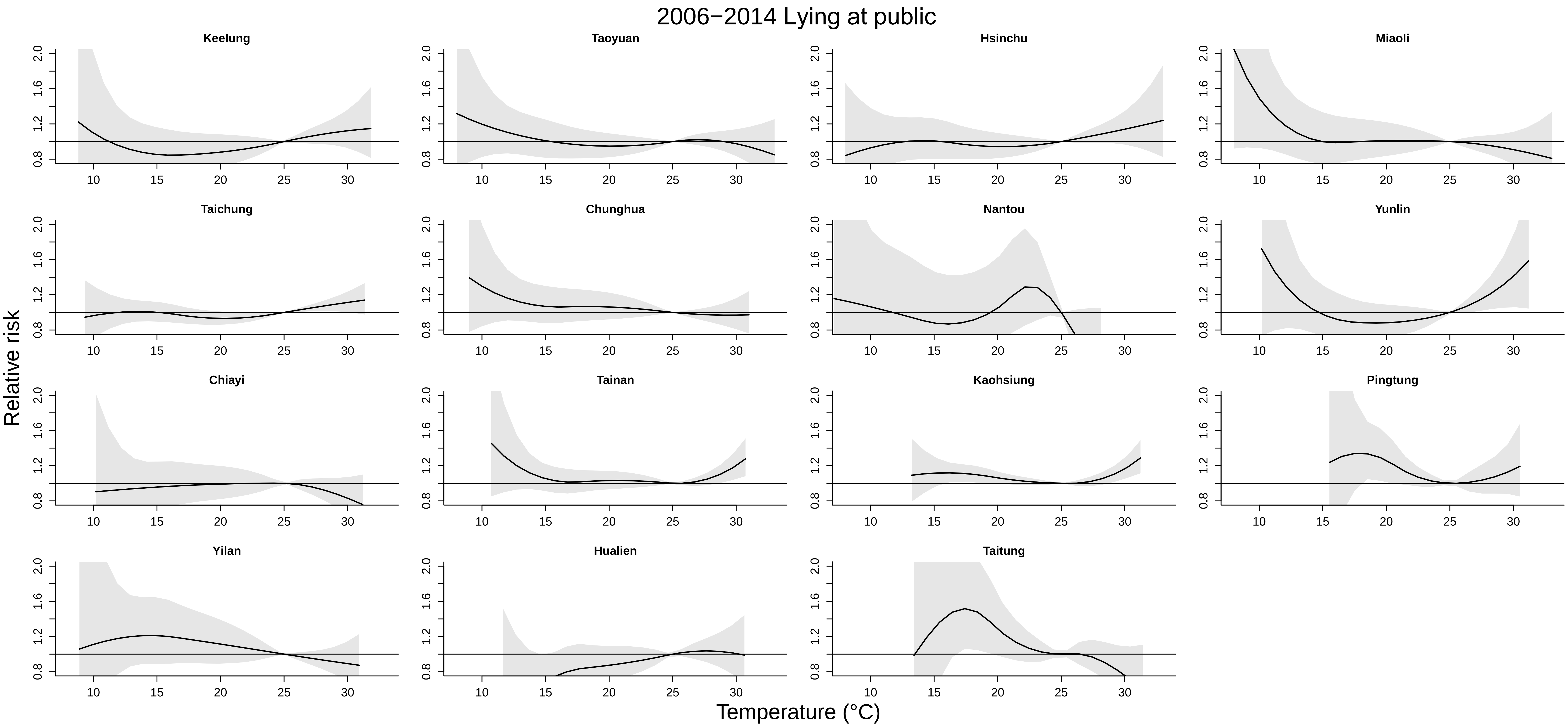

2006−2014 Lying at public
Keelung
Taoyuan
Hsinchu
Miaoli
Taichung
Chunghua
Nantou
Yunlin
Chiayi
Tainan
Kaohsiung
Pingtung
Yilan
Hualien
Taitung
Relative risk
Temperature (°C)

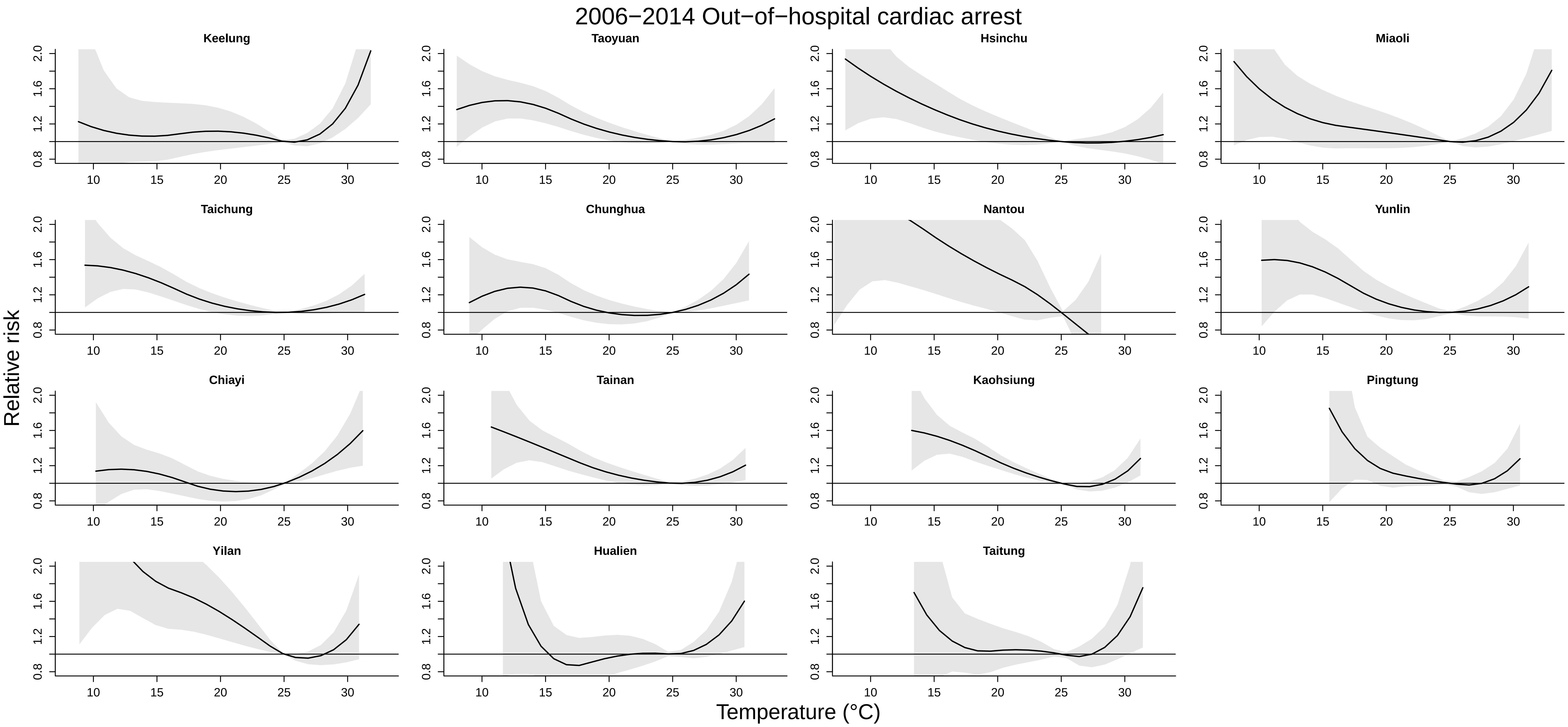

2006−2014 Out−of−hospital cardiac arrest
Keelung
Taoyuan
Hsinchu
Miaoli
Taichung
Chunghua
Nantou
Yunlin
Chiayi
Tainan
Kaohsiung
Pingtung
Yilan
Hualien
Taitung
Relative risk
Temperature (°C)

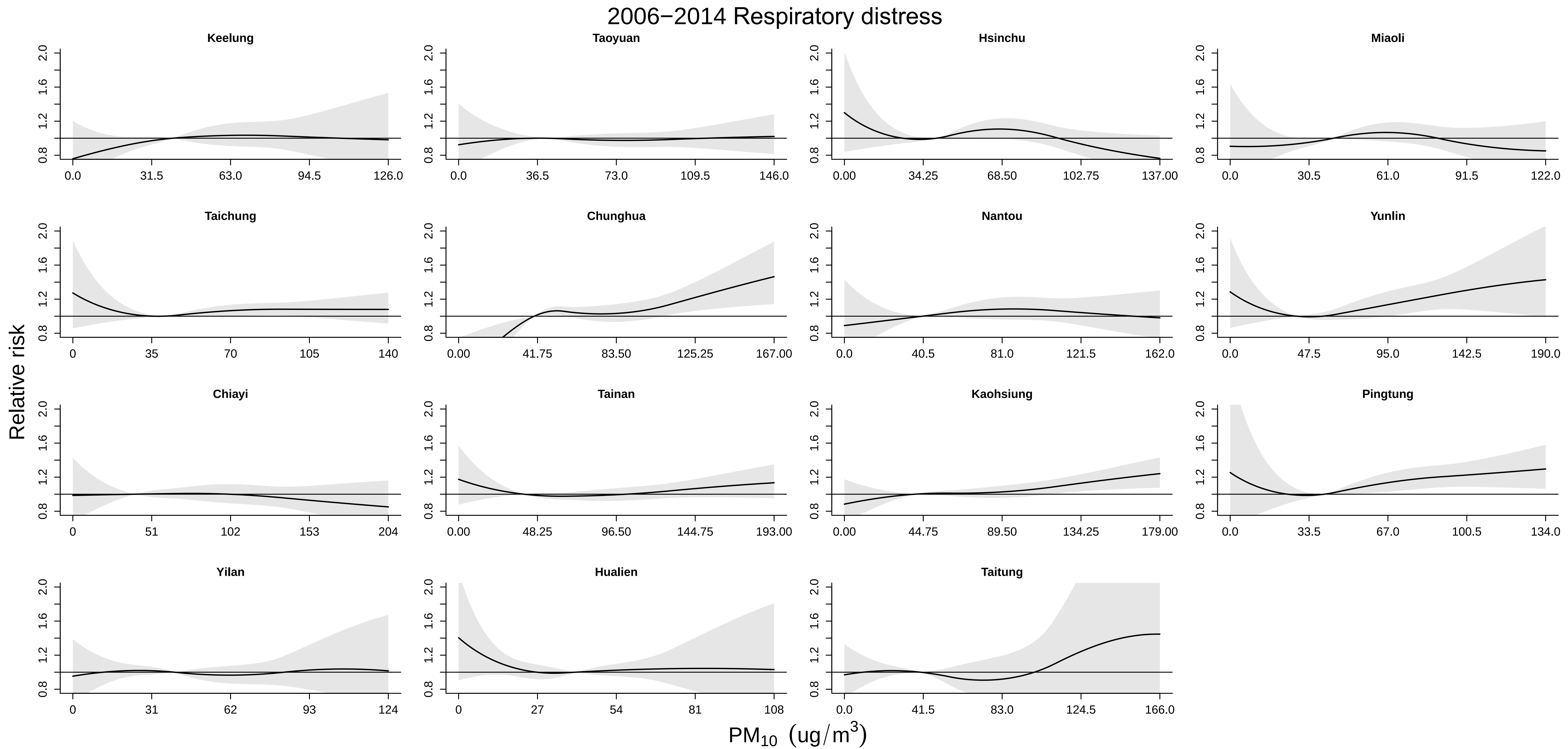
2006−2014 Respiratory distress
Keelung
Taoyuan
Hsinchu
Miaoli
Taichung
Chunghua
Nantou
Yunlin
Chiayi
Tainan
Kaohsiung
Pingtung
Yilan
Hualien
Taitung
Relative risk
PM_{10} (ug/m^3)

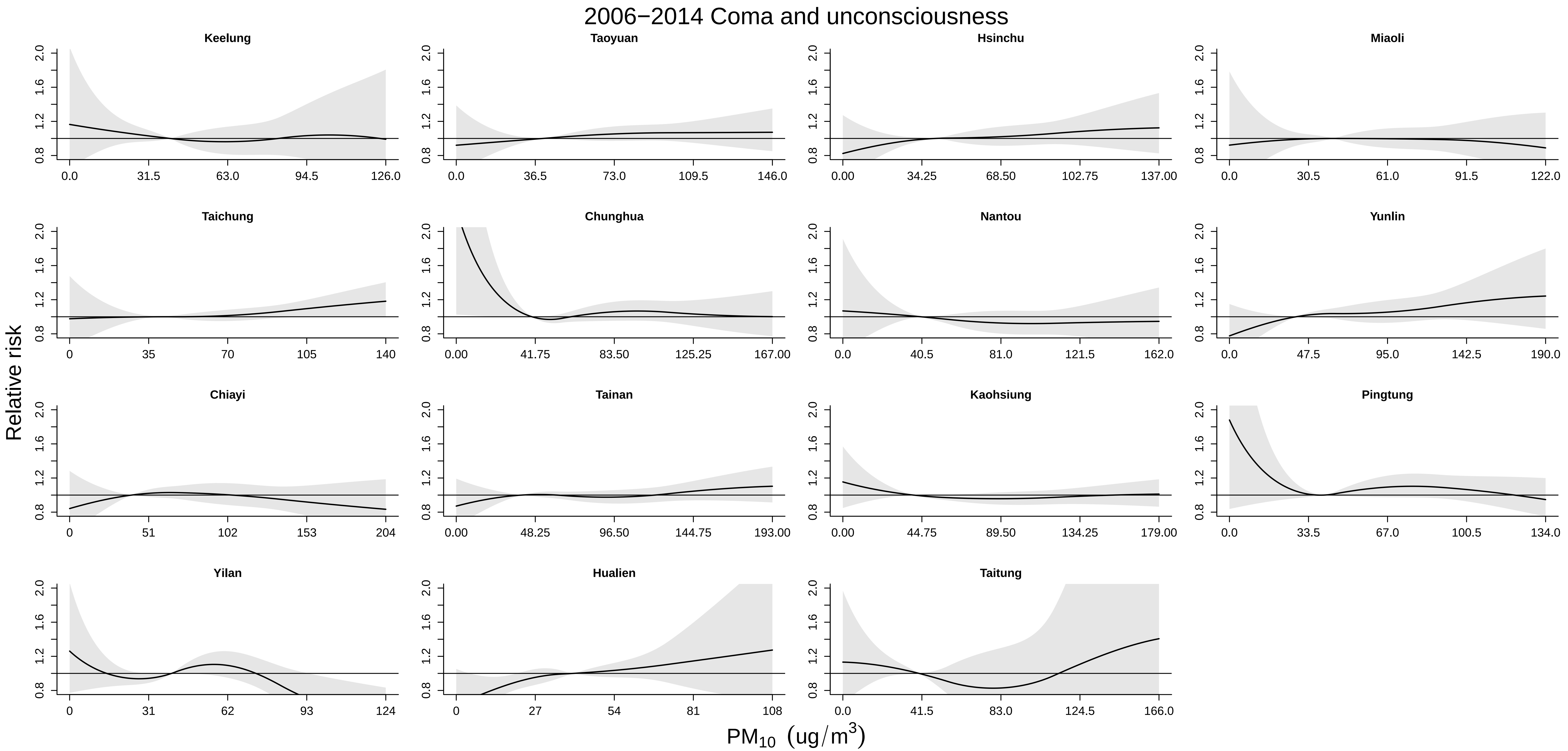

2006−2014 Coma and unconsciousness
Keelung
Taoyuan
Hsinchu
Miaoli
Taichung
Chunghua
Nantou
Yunlin
Chiayi
Tainan
Kaohsiung
Pingtung
Yilan
Hualien
Taitung
Relative risk
PM10 (ug/m3)
0.8
1.2
1.6
2.0
0.0 31.5 63.0 94.5 126.0
0.0 36.5 73.0 109.5 146.0
0.00 34.25 68.50 102.75 137.00
0.0 30.5 61.0 91.5 122.0
0 35 70 105 140
0.00 41.75 83.50 125.25 167.00
0.0 40.5 81.0 121.5 162.0
0.0 47.5 95.0 142.5 190.0
0 51 102 153 204
0.00 48.25 96.50 144.75 193.00
0.00 44.75 89.50 134.25 179.00
0.0 33.5 67.0 100.5 134.0
0 31 62 93 124
0 27 54 81 108
0.0 41.5 83.0 124.5 166.0

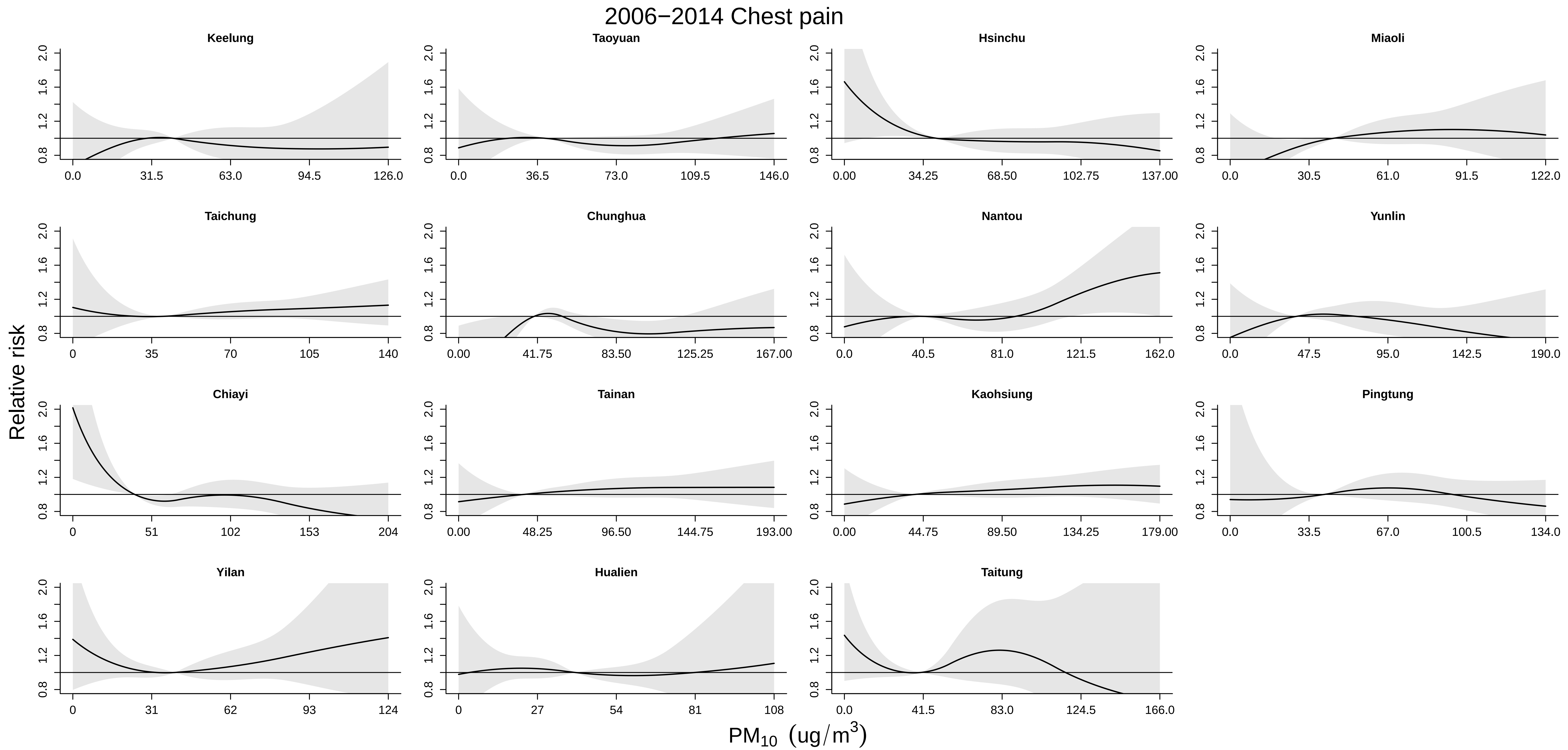
2006−2014 Chest pain
Keelung
Taoyuan
Hsinchu
Miaoli
Taichung
Chunghua
Nantou
Yunlin
Chiayi
Tainan
Kaohsiung
Pingtung
Yilan
Hualien
Taitung
Relative risk
PM10 (ug/m3)

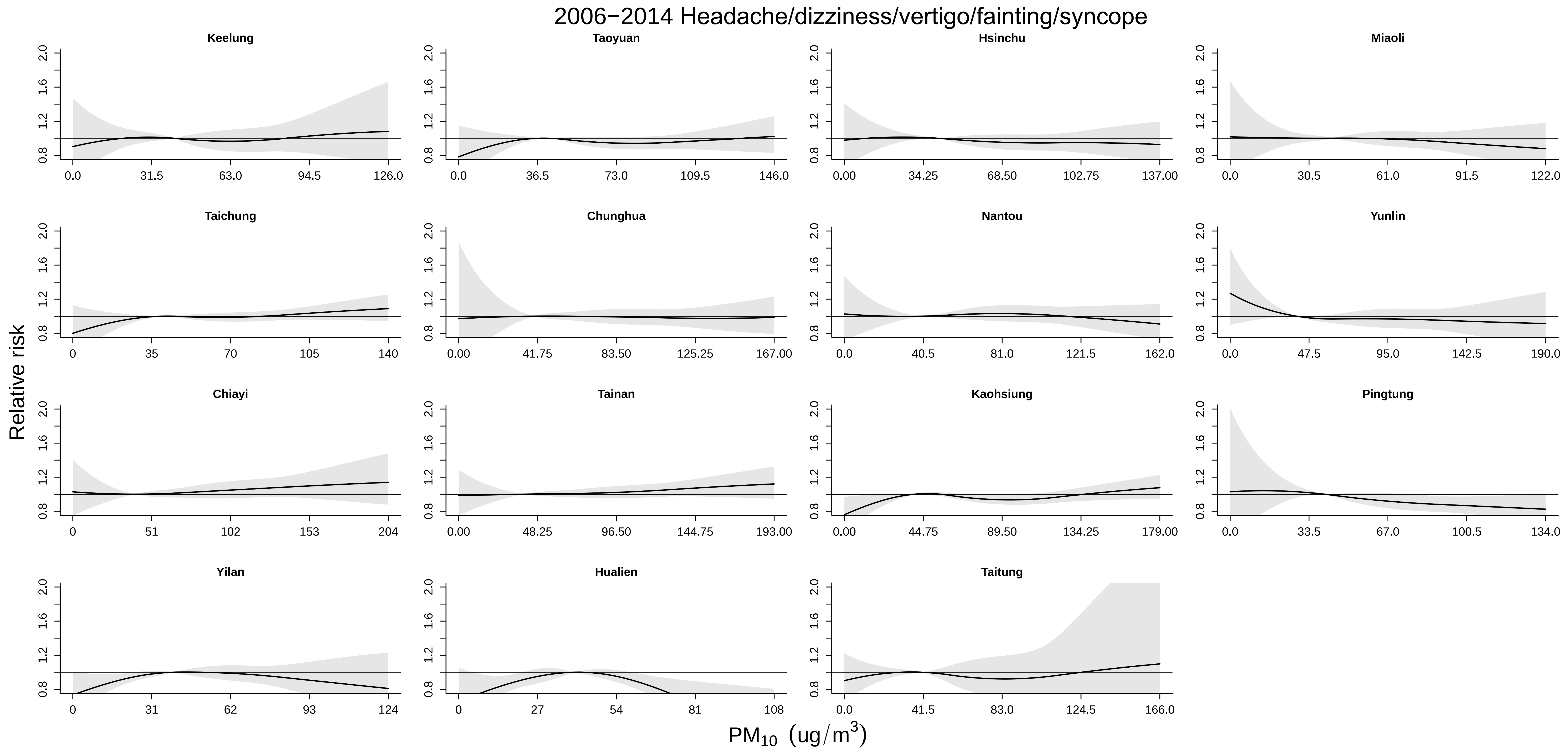
2006−2014 Headache/dizziness/vertigo/fainting/syncope
Keelung
Taoyuan
Hsinchu
Miaoli
Taichung
Chunghua
Nantou
Yunlin
Chiayi
Tainan
Kaohsiung
Pingtung
Yilan
Hualien
Taitung
Relative risk
PM_{10} (ug/m^3)

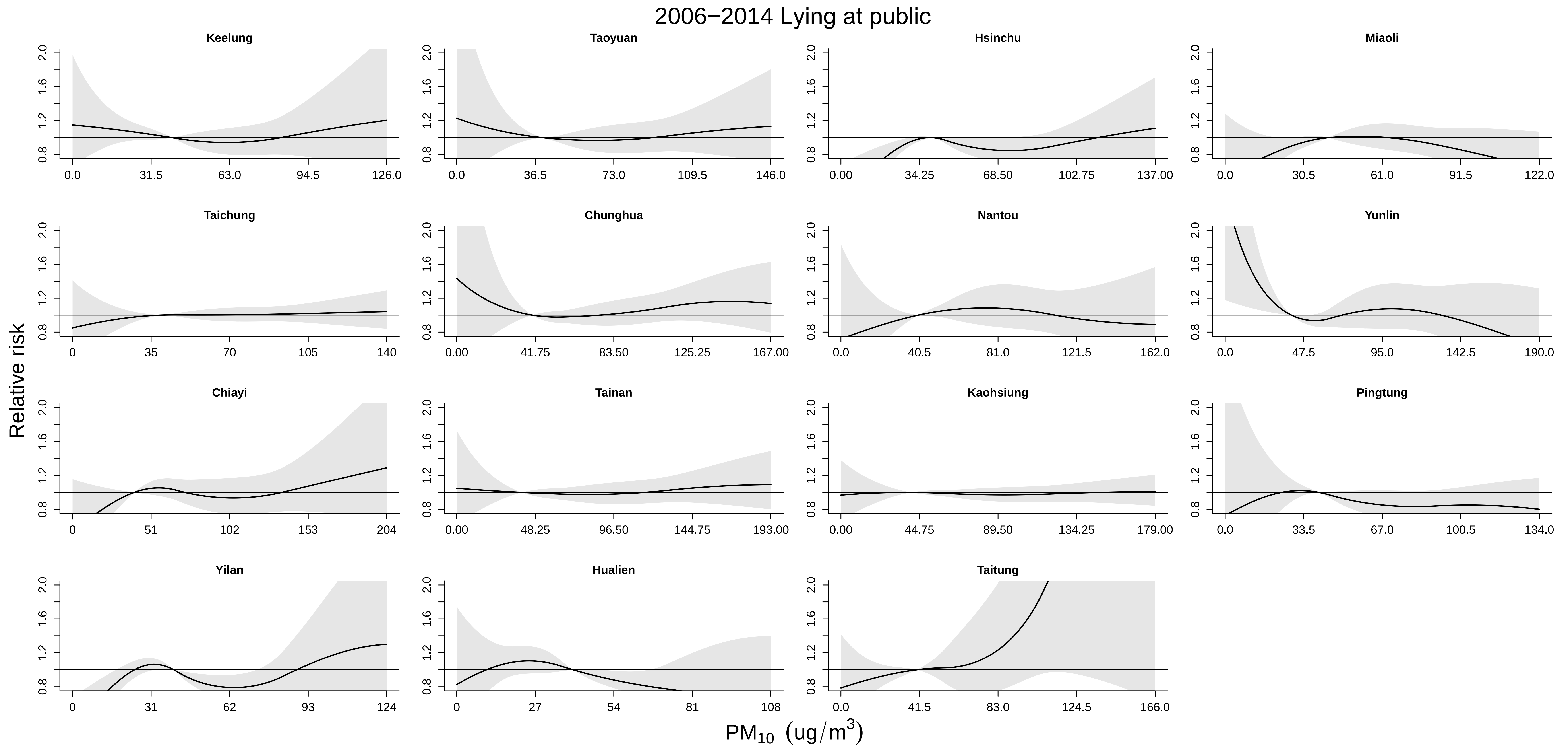
2006−2014 Lying at public
Keelung
Taoyuan
Hsinchu
Miaoli
Taichung
Chunghua
Nantou
Yunlin
Chiayi
Tainan
Kaohsiung
Pingtung
Yilan
Hualien
Taitung
Relative risk
PM_{10} (ug/m^3)

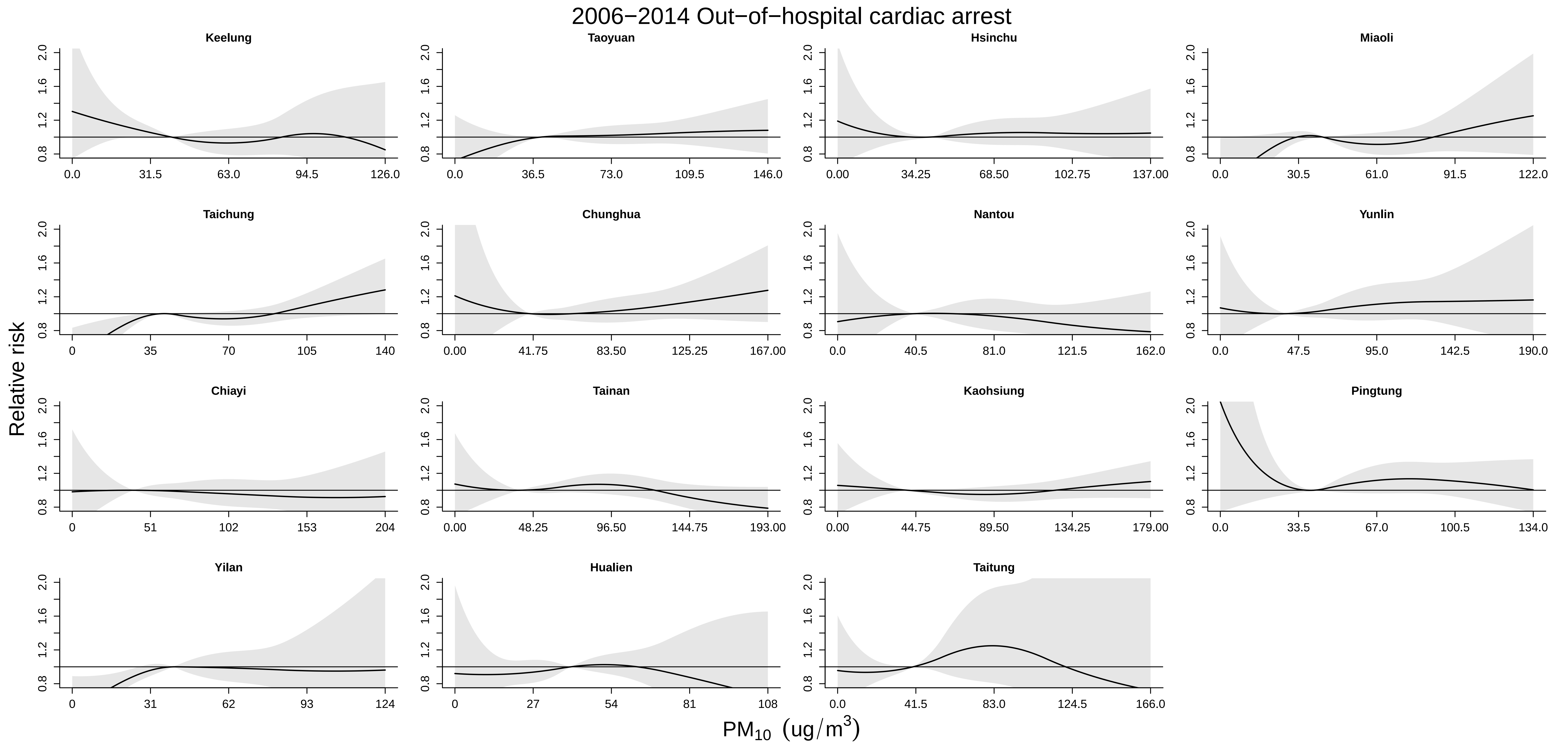

2006−2014 Out−of−hospital cardiac arrest
Keelung
Taoyuan
Hsinchu
Miaoli
Taichung
Chunghua
Nantou
Yunlin
Chiayi
Tainan
Kaohsiung
Pingtung
Yilan
Hualien
Taitung
Relative risk
PM10 (ug/m3)

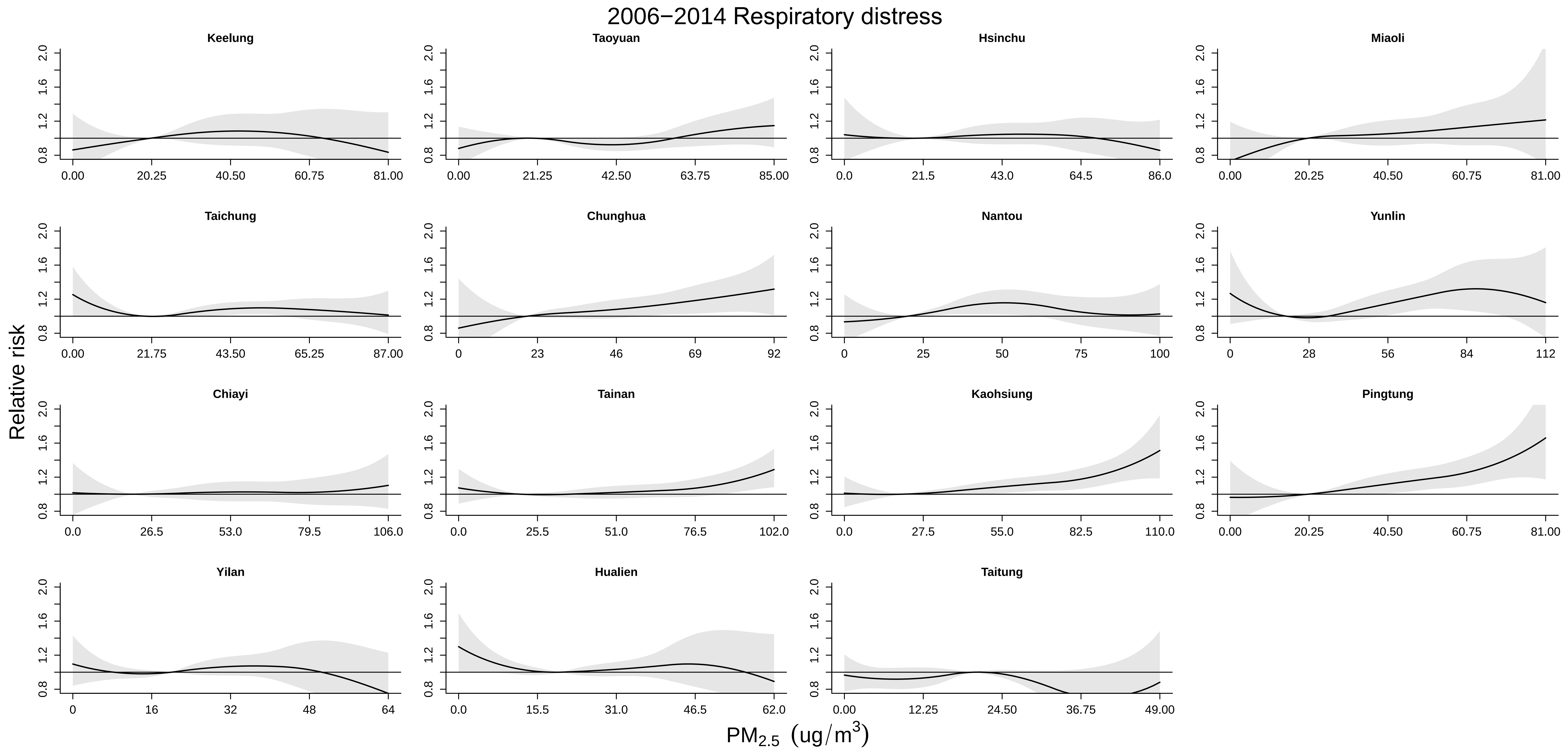
2006−2014 Respiratory distress
Keelung
Taoyuan
Hsinchu
Miaoli
Taichung
Chunghua
Nantou
Yunlin
Chiayi
Tainan
Kaohsiung
Pingtung
Yilan
Hualien
Taitung
Relative risk
$PM_{2.5}$ (ug/m^3)

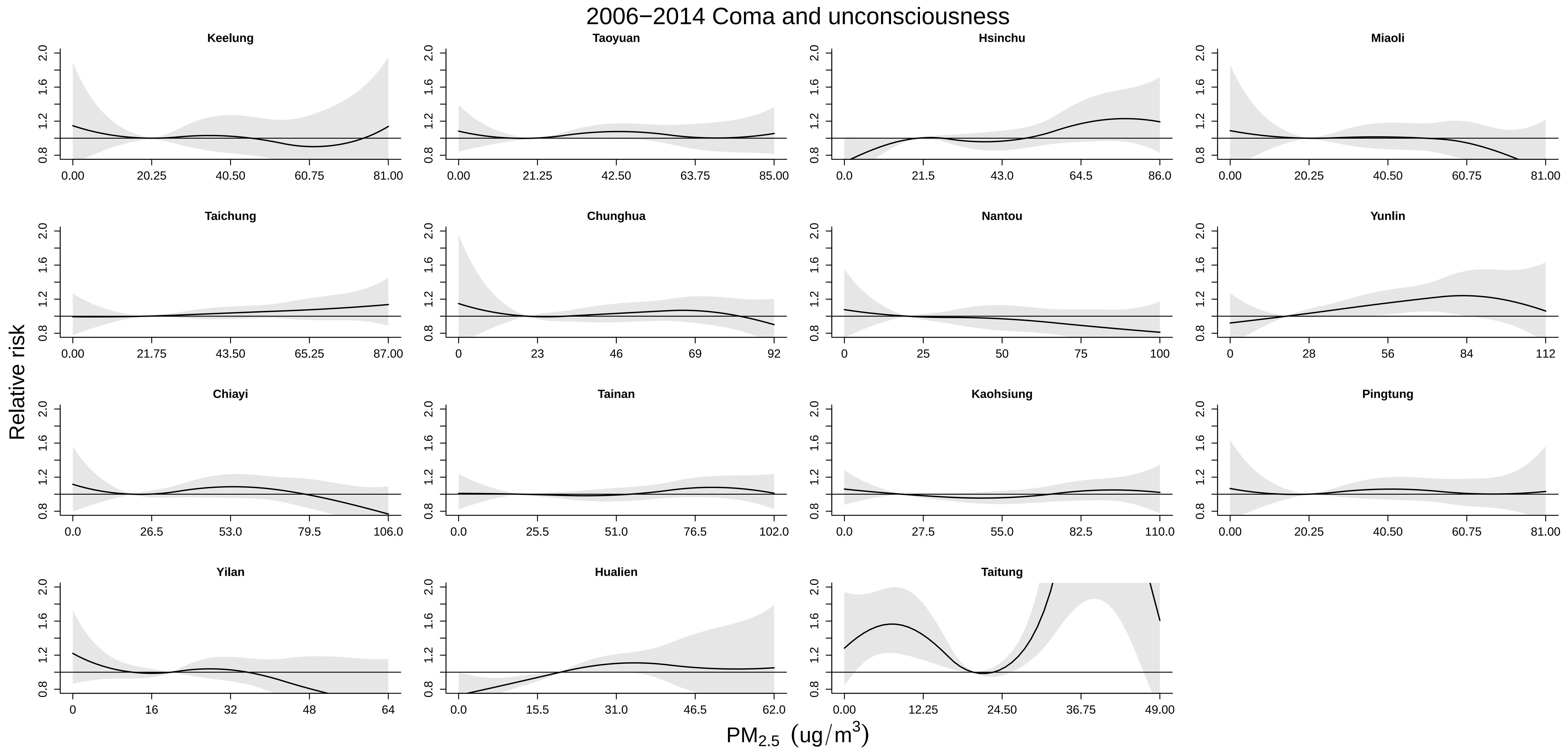

2006−2014 Coma and unconsciousness
Keelung
Taoyuan
Hsinchu
Miaoli
Taichung
Chunghua
Nantou
Yunlin
Chiayi
Tainan
Kaohsiung
Pingtung
Yilan
Hualien
Taitung
Relative risk
$PM_{2.5}$ (ug/m^3)

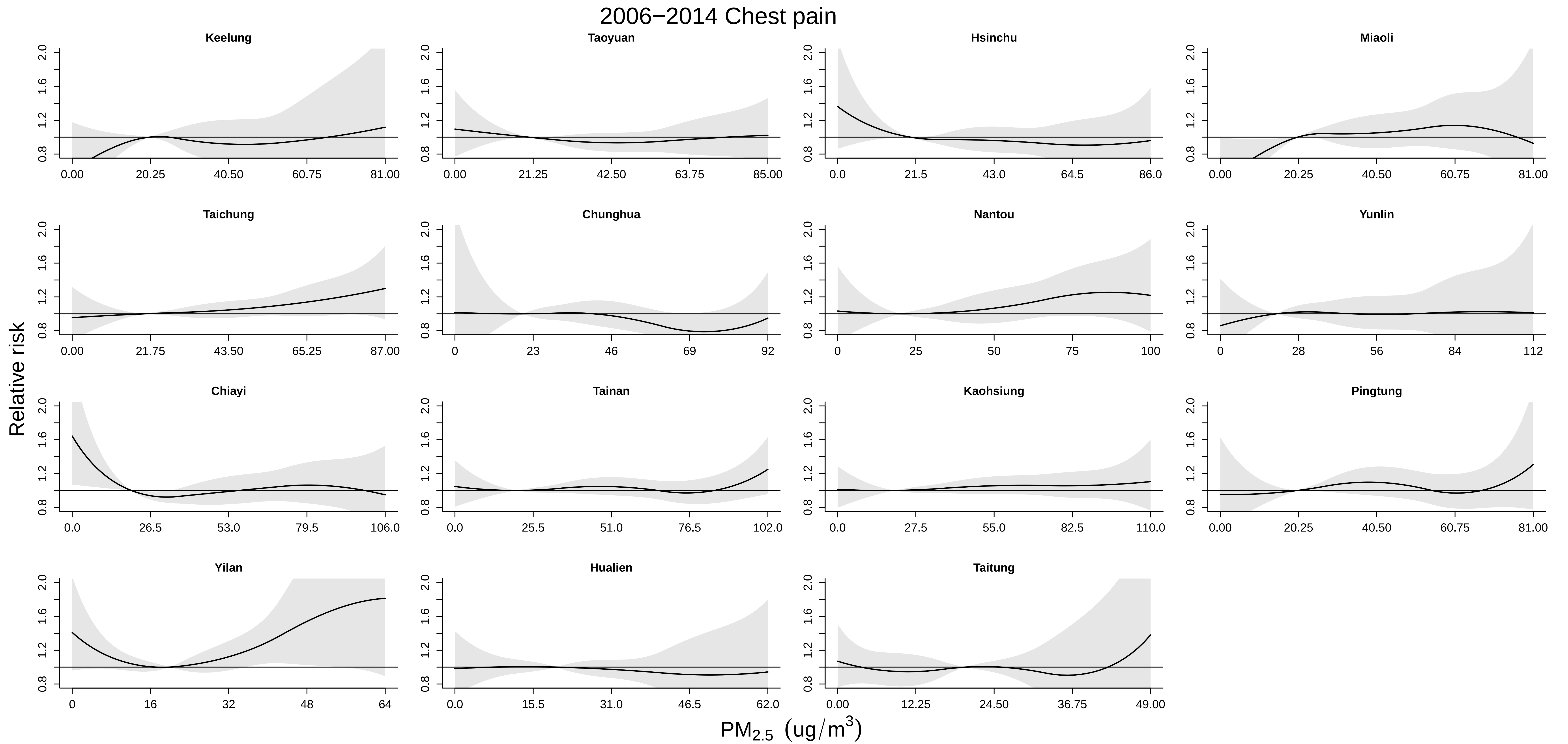

2006−2014 Chest pain
Keelung
Taoyuan
Hsinchu
Miaoli
Taichung
Chunghua
Nantou
Yunlin
Chiayi
Tainan
Kaohsiung
Pingtung
Yilan
Hualien
Taitung
Relative risk
PM2.5 (ug/m3)

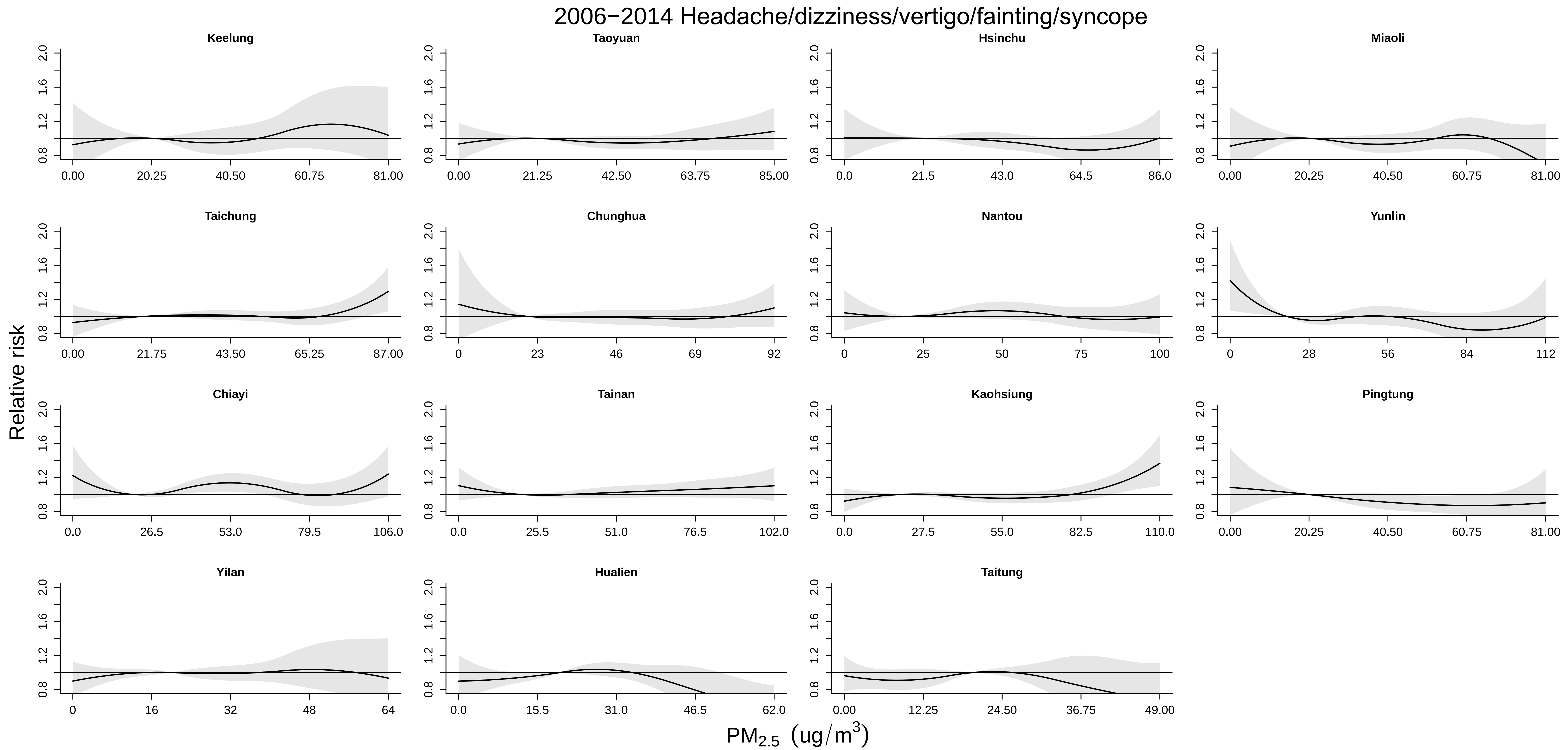

2006−2014 Headache/dizziness/vertigo/fainting/syncope
Keelung
Taoyuan
Hsinchu
Miaoli
Taichung
Chunghua
Nantou
Yunlin
Chiayi
Tainan
Kaohsiung
Pingtung
Yilan
Hualien
Taitung
Relative risk
$PM_{2.5}$ (ug/m^3)

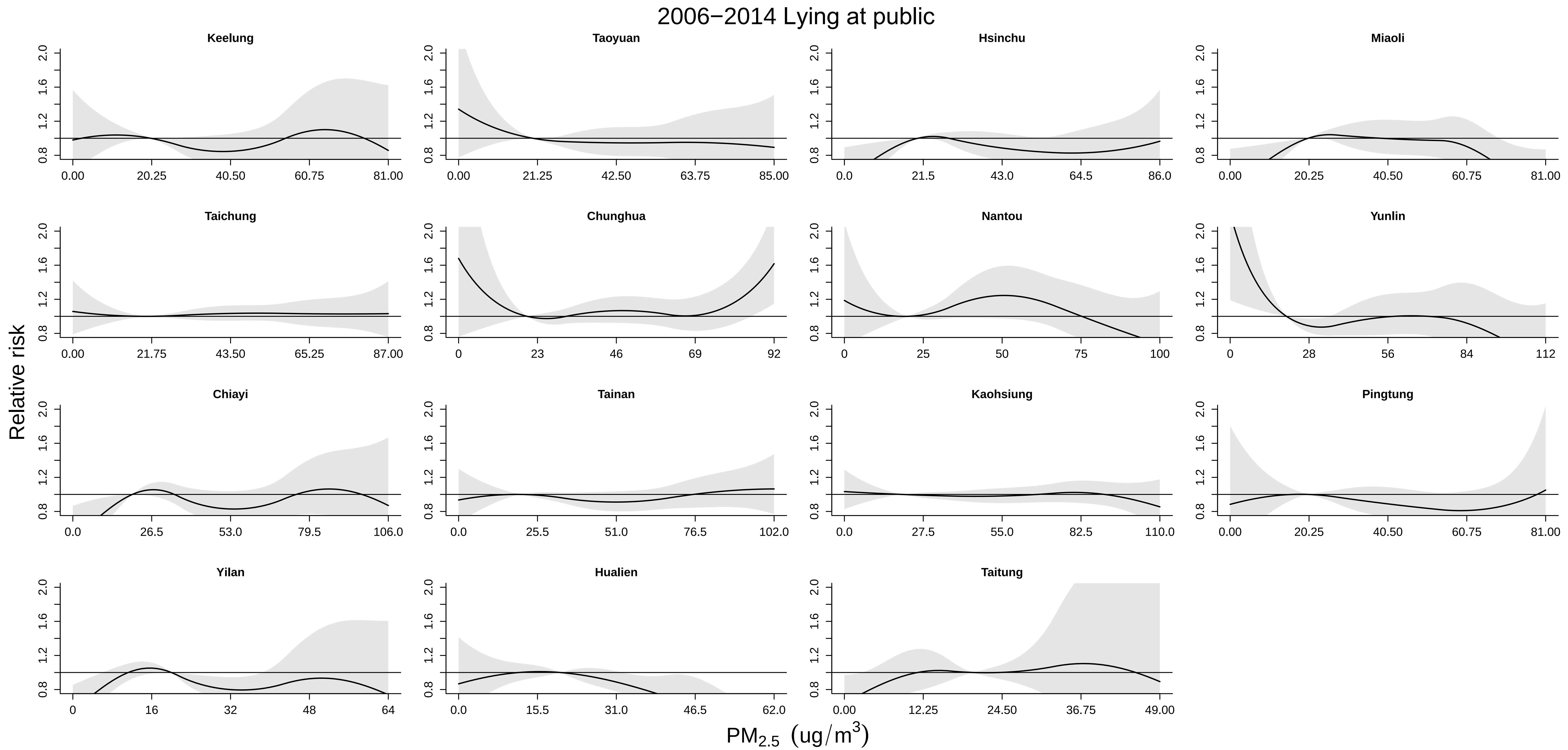
2006−2014 Lying at public
Keelung
Taoyuan
Hsinchu
Miaoli
Taichung
Chunghua
Nantou
Yunlin
Chiayi
Tainan
Kaohsiung
Pingtung
Yilan
Hualien
Taitung
Relative risk
PM2.5 (ug/m3)

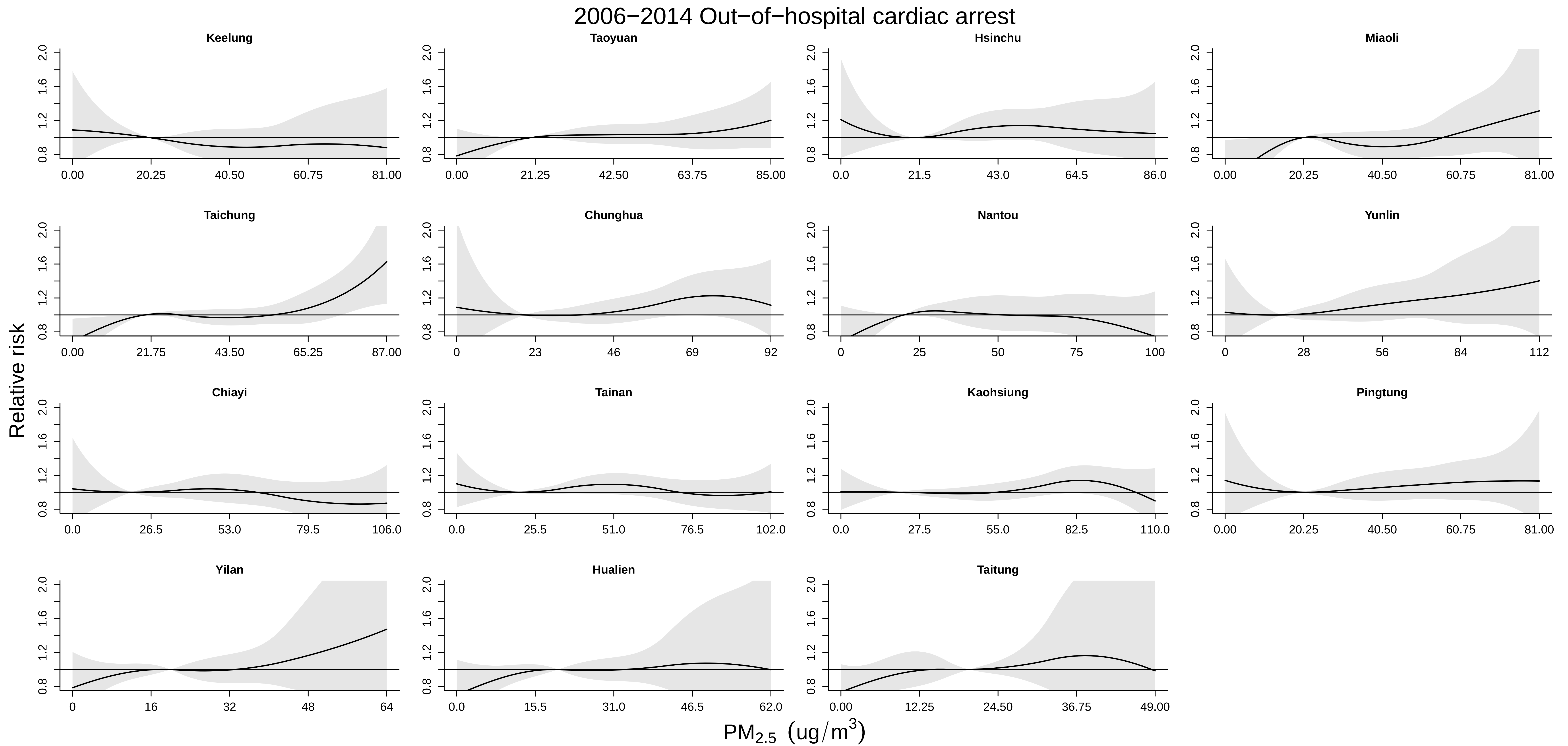

2006−2014 Out−of−hospital cardiac arrest
Keelung
Taoyuan
Hsinchu
Miaoli
Taichung
Chunghua
Nantou
Yunlin
Chiayi
Tainan
Kaohsiung
Pingtung
Yilan
Hualien
Taitung
Relative risk
0.8
1.2
1.6
2.0
0.00
20.25
40.50
60.75
81.00
21.25
42.50
63.75
85.00
0.0
21.5
43.0
64.5
86.0
21.75
43.50
65.25
87.00
0
23
46
69
92
25
50
75
100
28
56
84
112
26.5
53.0
79.5
106.0
25.5
51.0
76.5
102.0
27.5
55.0
82.5
110.0
16
32
48
64
15.5
31.0
46.5
62.0
12.25
24.50
36.75
49.00
$PM_{2.5}$ (ug/m³)
